# Robotic Rehabilitation in Spinal Cord Injury: Neurophysiological Basis and Severity-Based Clinical Framework

**DOI:** 10.3390/brainsci16070732

**Published:** 2026-07-11

**Authors:** Rocco Salvatore Calabrò, Andrea Calderone, Tiziana Di Gregorio, Maria Pia Onesta, Angelo Quartarone

**Affiliations:** 1 Scientific Institute for Research, Hospitalization and Healthcare (IRCCS) Centro Neurolesi Bonino Pulejo, 98124 Messina, Italy; roccos.calabro@irccsme.it (R.S.C.); angelo.quartarone@irccsme.it (A.Q.); 2 Unipolar Spinal Unit, Cannizzaro Hospital for Emergency Care, 95126 Catania, Italy; unispinale@ospedale-cannizzaro.it (T.D.G.); mp.onesta@gmail.com (M.P.O.)

**Keywords:** spinal cord injury, robotic rehabilitation, exoskeletons, neurorehabilitation, neuroplasticity, personalized rehabilitation, assistive technology, restorative rehabilitation

## Abstract

**Highlights:**

**What are the main findings?**

- Robotic rehabilitation in spinal cord injury (SCI) should be prescribed according to injury level, American Spinal Injury Association (ASIA) Impairment Scale (AIS) grade, residual function and goal hierarchy rather than device availability alone.
- The review proposes a severity-based and goal-oriented clinical framework that integrates upper limb robotics, lower limb exoskeletons, non-motor outcomes and advanced neurotechnologies.

**What are the implications of the main findings?**

- Personalized robotic prescription may improve alignment between patient priorities, realistic therapeutic targets and outcome monitoring.
- Future implementation should emphasize responder profiling, adaptive control, cost-effectiveness, home-based models and equitable access.

**Abstract:**

**Background/Objectives**: Spinal cord injury (SCI) causes heterogeneous motor, sensory, autonomic, and participation limitations; recovery priorities vary by injury level, completeness, time since injury and residual function. Robotic rehabilitation has expanded from assistive technology to restorative, compensatory and health-promoting interventions, but patient-tailored prescription frameworks remain underdeveloped. **Methods**: PubMed/MEDLINE was searched from database inception to May 2026 using predefined domain-specific strategies, and findings were synthesized narratively to integrate mechanistic, clinical, safety and implementation evidence. **Results**: Robotic systems can increase task-specific repetition, sensorimotor feedback, active engagement and quantitative monitoring. Upper-limb robotics are feasible in cervical SCI and may support reach, grasp and activities of daily living, although SCI-specific controlled evidence remains limited. Lower-limb exoskeletons and locomotor robots can support gait practice, upright mobility, exercise exposure and selected secondary health outcomes, but walking speed, energy expenditure, cost, supervision needs and community translation remain important barriers. Sensory and non-motor effects, including proprioceptive input, spasticity, pain, bowel routine, cardiometabolic conditioning, participation and psychological well-being, are clinically relevant but should be interpreted according to evidence strength. Robotics combined with functional electrical stimulation, virtual reality, brain–computer interfaces, non-invasive brain stimulation and artificial intelligence-driven adaptation is promising but not yet routine. **Conclusions**: Robotic rehabilitation in SCI should be prescribed through a severity-based process that considers lesion level, American Spinal Injury Association Impairment Scale grade, residual voluntary and sensory function, safety, patient priorities and measurable goals. The proposed framework supports transparent selection and prospective validation of individualized robotic rehabilitation and shifts decisions beyond device availability toward clinically meaningful and equitable implementation.

## 1. Introduction

Spinal cord injury (SCI) remains one of the most complex conditions in neurological rehabilitation because it combines motor paralysis, sensory impairment, autonomic dysfunction, secondary complications (including psychological issues) and long-term restrictions in participation. The global incidence and burden remain substantial, and the neurological impairment profile ranges from near-complete loss of voluntary control to selective motor deficits with or without preserved sensory pathways [1,2]. This heterogeneity is not only anatomical. It shapes priorities, expectations and the meaning of recovery. The International Standards for Neurological Classification of Spinal Cord Injury and the American Spinal Injury Association (ASIA) Impairment Scale (AIS) have improved the language used to describe neurological severity, but these tools do not automatically indicate which rehabilitation treatment should be prescribed [3,4]. The gap between classification and treatment selection is one of the reasons why robotic rehabilitation still varies widely across centers.

Patient-reported priorities have repeatedly shown that recovery goals differ according to injury level and residual function. Restoration of arm and hand function is consistently rated as a dominant priority by individuals with tetraplegia, whereas walking, bowel and bladder function, sexual function, pain relief and independence remain central for many individuals with paraplegia [5,6,7,8]. These preferences are clinically important because they challenge a single-device view of robotic rehabilitation. A wearable exoskeleton may be transformative for upright exercise in one person and largely irrelevant for another person whose main barrier to independence is hand dexterity. In the same way, an upper limb robot may provide high-dose reaching practice in incomplete cervical SCI but may have a more compensatory or assistive role in severe motor-complete tetraplegia. Personalized robotic prescription should therefore begin with the person, not the machine.

Robotics entered neurorehabilitation mainly as a means of reducing therapist workload and delivering reproducible movement. The field has since evolved toward a broader therapeutic vision. Robotic systems can now support intensive task-specific practice, quantify performance, modulate assistance, augment feedback and integrate with neurotechnologies such as functional electrical stimulation, virtual reality, brain–computer interfaces and non-invasive brain stimulation [9,10,11,12]. This evolution has created justified interest in restorative effects, particularly in incomplete SCI where spared descending pathways can be recruited by high-intensity sensorimotor training. Yet the same enthusiasm has sometimes outpaced clinical reasoning. Device availability, commercial visibility and institutional investment may drive prescription more than injury severity, goal hierarchy or response monitoring. A framework that explicitly links neurological profile to realistic robotic goals is needed.

Previous reviews have examined rehabilitation robotics in SCI and broader neurological populations, including upper-limb robotic therapy, lower-limb exoskeletons, robot-assisted gait training (RAGT), exoskeleton-assisted walking and hybrid neurotechnologies [9,10,11,12,13,14]. However, many syntheses are organized primarily around devices or mixed neurological cohorts, and findings from stroke rehabilitation are not always directly transferable to SCI because lesion level, sensory preservation, autonomic dysfunction, musculoskeletal loading, hand priorities and long-term participation barriers differ substantially. Important gaps therefore remain in translating robotic mechanisms into clinically usable prescription rules. This review addresses those gaps by integrating neurophysiological basis, technology taxonomy, motor, sensory and non-motor outcomes, implementation constraints and a severity-based framework for robotic prescription in SCI.

A second reason for a personalized framework is that SCI rehabilitation increasingly spans hospital, outpatient, community and home environments. The same robotic strategy may have a different value at each stage. During inpatient rehabilitation, a device may create safe repetition and early verticalization. Later, it may become a way to preserve conditioning, practice complex tasks or support participation. Time since injury also modifies expectations because spontaneous neurological recovery, compensatory learning and secondary complications contribute differently over time.

This review aims to (i) summarize the neurophysiological basis of robotic rehabilitation in SCI; (ii) review current upper- and lower-limb robotic technologies; (iii) synthesize evidence for motor, sensory and non-motor outcomes; (iv) discuss combined approaches with advanced neurotechnologies; and (v) propose a severity-based and goal-oriented clinical prescription framework. The framework is intended to support transparent clinical reasoning rather than to replace individualized multidisciplinary judgment.

### Search Strategy and Narrative Synthesis

Although this article is structured as a narrative review, a structured PubMed/MEDLINE search was performed from database inception to May 2026 using predefined domain-specific strategies. The search was intentionally designed to cover the main SCI profiles relevant to robotic rehabilitation, including tetraplegia, paraplegia, cervical SCI, thoracic/lumbar SCI and both complete and incomplete injuries. Search terms and selection procedures are reported in Supplementary Table S1, and a domain-level evidence map is provided in Supplementary Table S2. A narrative approach was chosen because the review addresses a clinically integrative objective: to connect mechanisms, device classes, candidate populations, safety screening, outcomes and implementation issues across highly heterogeneous technologies and study designs. This scope was not suited to a single pooled quantitative estimate. Accordingly, no formal risk-of-bias assessment or PRISMA flow diagram was performed. This design allows broad clinical synthesis but may introduce selection bias and limits the strength of causal inferences.

## 2. Neurophysiological Rationale of Robotics in SCI

The neurophysiological basis of robotic rehabilitation in SCI is grounded in activity-dependent plasticity, repeated task execution and sensory feedback. Neural circuits below the lesion are not passive targets; they can generate patterned motor output when appropriate afferent input, descending drive or neuromodulatory facilitation is available [15,16]. The central pattern generator (CPG) provides one example of spinal circuitry capable of producing coordinated rhythmic output for walking independently of direct cortical command [16]. After SCI, repeated sensory feedback during robot-assisted stepping may help modulate locomotor networks, strengthen spared connections and support adaptive reorganization. Robotic gait interventions are therefore best understood as structured sensorimotor stimuli, not simply as mechanical mobility devices.

Robotic devices can help structure this input by providing large numbers of consistent, goal-directed movements that would be difficult to reproduce manually with the same intensity and kinematic precision. The core therapeutic principle is not the robot itself, but the opportunity to expose the nervous system to meaningful sensorimotor contingencies at a sufficient dose.

Assist-as-needed control is clinically important because motor learning depends on practice that is specific, salient and sufficiently challenging. Robotic systems can deliver high-repetition stepping, reaching or grasping while maintaining alignment, safety and feedback. Assistance can be calibrated so that the person contributes as much voluntary effort as possible while the device prevents complete task failure. These paradigms may reduce learned passivity compared with fully passive guidance, although the optimal assistance level depends on impairment severity, fatigue, spasticity and task demands [17,18,19].

Sensorimotor feedback is a second core mechanism. Proprioceptive input, load-related afference, cutaneous feedback and visual feedback converge on spinal and supraspinal networks. In lower limb robotics, repeated stepping with body-weight support may activate spinal locomotor networks and CPG-like circuits [20,21,22]. In upper limb robotics, visuomotor and proprioceptive feedback may support error correction, body-schema updating and motor planning. Making performance visible can also reinforce attention and motivation, which are relevant modulators of rehabilitation learning.

Neuroplasticity after SCI occurs at multiple levels. Cortical representations may reorganize after deafferentation and disuse, while corticospinal excitability, intracortical inhibition and interhemispheric balance may change during recovery or chronic adaptation [23,24]. Recent systematic evidence further supports the view that SCI is associated with distributed cortical and subcortical plastic changes that may interact with rehabilitation-induced sensorimotor experience [25]. Robotic training can interact with these processes by increasing the amount of attempted movement, coupling intention with sensory consequences and providing quantitative feedback. In incomplete SCI, spared descending pathways may be strengthened through repeated voluntary engagement. In motor-complete SCI, restorative claims should be more cautious, but repeated afferent activation and upright loading may still influence spinal excitability, autonomic regulation, musculoskeletal health and embodiment.

Embodiment and agency are also relevant to robotic rehabilitation. Standing or walking in an exoskeleton, manipulating objects with a hand robot or seeing a virtual limb move in response to motor intention may modify the perceived relationship between the body and the environment [26]. Body ownership, agency and multisensory integration influence motor planning and engagement. Systems that couple intention, action and feedback may therefore be more therapeutic than devices that only move the limb passively, especially in BCI-linked or virtual reality-supported protocols.

**Figure 1:**
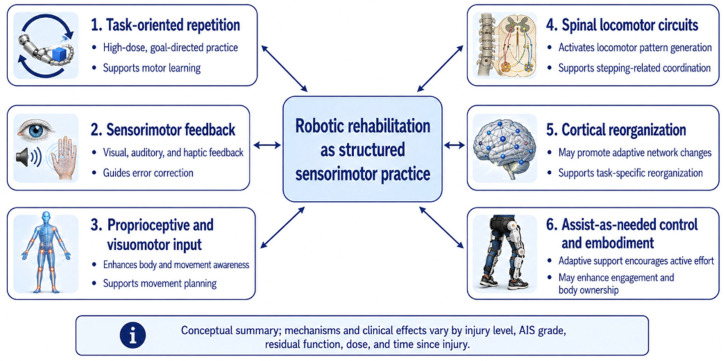
Key neurophysiological concepts relevant to robotic rehabilitation in spinal cord injury. The figure summarizes concepts commonly discussed in the robotic rehabilitation literature, including task-oriented repetition, sensorimotor feedback, spinal locomotor networks, cortical reorganization, embodiment and assist-as-needed control.

Severity determines how these mechanisms should be interpreted. A person with AIS D paraplegia and preserved hip flexion requires a different dose, assistance level and outcome hierarchy from a person with AIS A thoracic SCI using an exoskeleton primarily for standing, exercise and participation. Similarly, incomplete cervical SCI may justify high-repetition reach-to-grasp training, whereas severe tetraplegia may require assistive hand technology for activities of daily living. Figure 1 summarizes key neurophysiological concepts commonly invoked in robotic rehabilitation, including task-oriented practice, sensorimotor feedback, spinal locomotor circuitry, cortical reorganization, embodiment and assist-as-needed control. It should be interpreted as a conceptual map rather than as evidence of a single mechanistic pathway [27,28].

Timing and dose further influence the biological plausibility of robotic therapy. Early after SCI, circuits may be particularly responsive to patterned activity, although medical instability, pain, orthostatic intolerance and skin vulnerability may restrict the safe intensity of training. In the chronic stage, plasticity remains possible but usually requires stronger task salience, sufficient repetition and careful avoidance of maladaptive compensation. Robotic devices are useful when they transform an otherwise brief therapeutic attempt into a measurable training episode with controlled assistance and feedback. The relevant dose should not be described only as minutes on a device. It should also include active repetitions, effort, error, feedback frequency, progression, rest, autonomic load and transfer to unassisted tasks.

The distinction between passive movement and active intention is equally important. Movement generated without attention or voluntary drive may provide range of motion and sensory input, but it is less likely to drive task-specific learning than training that requires anticipation, error detection and correction. For this reason, modern robotic protocols increasingly emphasize active participation, adaptive resistance and meaningful goals. Electromyographic triggering, impedance control, virtual tasks and therapist-selected difficulty can help align assistance with residual capacity. These features are especially relevant in incomplete SCI, where the therapeutic objective is to amplify residual control rather than replace it. A severity-based framework should therefore ask not only whether a robot can move the limb, but whether the patient can participate in the movement in a way that matches the intended mechanism.

## 3. Upper Limb Robotics in SCI

### 3.1. Clinical Goals in Tetraplegia

Upper limb function is the decisive determinant of independence for many individuals with cervical SCI. Small gains in shoulder control, elbow extension, wrist stabilization, hand opening or grasp timing can change the ability to eat, groom, use a phone, operate a wheelchair, transfer, write or manage personal care. This explains why rehabilitation goals in tetraplegia are often highly specific. A person may not need global arm recovery to experience meaningful functional change. The ability to bring the hand to the mouth, stabilize the wrist during a grasp or release an object reliably can have a disproportionate effect on autonomy and caregiver burden [29,30]. Robotic upper limb therapy should therefore be evaluated not only by impairment scales, but also by whether it improves activities that matter in daily life.

Clinical goal setting should begin with neurological level, residual voluntary control, sensibility and compensatory strategies. Individuals with incomplete cervical SCI may be candidates for restorative training aimed at selective strengthening, coordination and dexterity. Those with more severe motor loss may require technology that provides movement assistance, enables repetitive practice despite weakness or supports compensatory activities of daily living. The distinction is not absolute. Even assistive robots can provide therapeutic practice if the person actively attempts the task, and even restorative systems may serve compensatory goals by improving endurance or movement confidence. For this reason, upper limb robotic prescription in tetraplegia should be framed around reach, grasp, release, manipulation, bimanual coordination and task integration.

Clinical goal setting in tetraplegia should also consider the interaction between proximal control and distal dexterity. A person who cannot stabilize the shoulder or elbow may fail to use a technically preserved grasp during real activities, while a person with good proximal control but poor finger opening may need a distal hand device rather than a large workspace robot. Robotic assessment can be helpful because it quantifies movement smoothness, workspace, speed, force and compensatory trunk motion. These metrics should be interpreted alongside clinical observation and patient-selected activities. A therapy target such as feeding requires reaching, wrist orientation, grasp timing, endurance and object manipulation, so a device that trains only one component may need to be combined with conventional occupational therapy.

### 3.2. Main Robotic Systems

Several robotic systems have been studied or adopted for upper limb rehabilitation, although SCI-specific evidence is far less extensive than stroke evidence. Representative upper limb platforms should be distinguished according to the degree of robotic assistance, the limb segment targeted, the residual voluntary function required and the intended therapeutic mechanism. Armeo Spring and Armeo Power are not clinically equivalent: the former primarily supports gravity-assisted task-oriented practice when residual proximal movement is present, whereas the latter provides powered assistance when active movement is severely limited and the target is assisted mobilization or active-assisted practice. ALEX-type and Diego systems mainly address proximal arm support, bilateral practice or workspace exploration, while Amadeo, Hand of Hope and Gloreha or soft glove systems are more distal or hand-focused. End-effector platforms such as InMotion and ReoGo provide guided, quantifiable reaching trajectories. Table 1 provides a comparative clinical summary and highlights why device selection should be driven by target function, residual voluntary control and intended mechanism rather than by device availability alone [10,28,31,32,33,34].

The most important prescription question is not which device is technologically superior. It is which device matches the target function, the available residual movement and the expected mechanism of improvement. A shoulder-elbow robot may be poorly matched to a patient whose main goal is pinch release. A hand exoskeleton may be insufficient for someone who cannot position the arm in space. An electromyography-triggered device may be useful when voluntary activation is present but too weak to produce functional motion. A gravity-supported exoskeleton may be preferable when proximal weakness limits repetition. The clinical reasoning must also consider pain, spasticity, joint range, skin integrity, fatigue and the ability to understand feedback.

The main upper limb platforms differ more in therapeutic logic than in brand identity. Exoskeleton or gravity-supported systems can enlarge the reachable workspace and enable repeated functional tasks. End-effector devices may standardize trajectories and quantify kinematics with high precision. Electromyography-triggered hand systems link intention to grasp assistance and may be particularly attractive when small voluntary signals are present. Soft gloves and portable hand robots are still evolving, but they address a clinically important gap between laboratory training and daily use. Table 1 summarizes representative systems and highlights why device selection should follow the impaired task, the available voluntary signal and the desired therapeutic endpoint.

Clinical availability should be distinguished from research maturity. Some platforms listed in Table 1 are used in specialized rehabilitation services or routine technology-enabled therapy programs, whereas other systems remain mainly within pilot studies, device-specific research protocols or highly specialized laboratories. Availability also varies by country, reimbursement pathway, staff training and maintenance infrastructure. This distinction is important because evidence derived from a research-grade system may not automatically translate into routine clinical delivery, and evidence from stroke cohorts should be extrapolated to cervical SCI only when the underlying motor, sensory and functional constraints are comparable.

### 3.3. Current Evidence

Current evidence for upper limb robotics in SCI is encouraging but not definitive. Early studies with Armeo Spring and other devices showed feasibility, safety and possible gains in arm function in subacute cervical SCI [35,36]. Chronic SCI studies have reported improvements in kinematics, movement quality and selected functional outcomes, but sample sizes are small and many designs lack control groups [37,38]. Trials using assist-as-needed robotic therapy suggest that adaptive assistance is feasible, although superiority over other intensive approaches is not established [39]. Pilot randomized evidence in tetraplegia indicates that robotic therapy can be comparable or additive to conventional occupational therapy, but robust conclusions about long-term activities of daily living remain limited [40,41].

The field is constrained by heterogeneity. Studies include different injury levels, AIS grades, time since injury, devices, training intensities and outcomes. Some trials focus on impairment-level measures, while others emphasize task performance, self-perception or independence. Follow-up is often short, which makes it difficult to know whether gains persist or translate into home activities. Another limitation is that many upper limb robotic technologies were developed for stroke, then adapted to SCI. Cervical SCI has distinct patterns of sensory impairment, autonomic instability, hand intrinsic weakness, tenodesis use and compensatory strategies. Future trials should therefore be SCI-specific and should include outcomes such as the Graded Redefined Assessment of Strength, Sensibility and Prehension, Spinal Cord Independence Measure, goal attainment, device-derived kinematics, participation and caregiver time [42,43,44].

## 4. Lower Limb Robotics and Exoskeletons

### 4.1. Restorative Versus Assistive Gait Robotics

Lower limb robotic rehabilitation in SCI includes a wide spectrum of approaches that should not be treated as interchangeable. A wearable exoskeleton used for overground standing and walking has a different clinical purpose from a treadmill-based robotic orthosis used to deliver repetitive stepping with body-weight support. The first may function as assistive mobility, therapeutic exercise or a participation tool. The second is more often used as a restorative gait training platform or as a way to provide task-specific locomotor input during inpatient or outpatient rehabilitation. The distinction between assistive and restorative robotics is therefore a clinical distinction, not simply a technical one [45,46].

**Figure 2:**
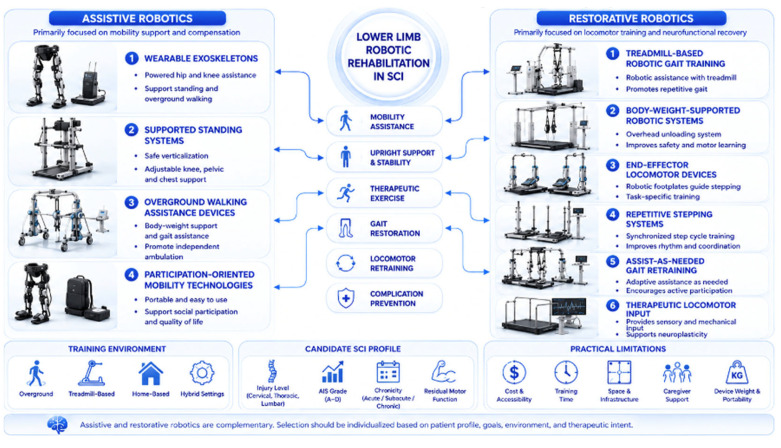
Taxonomy of assistive and restorative lower-limb robotic approaches in spinal cord injury. The figure distinguishes assistive mobility, verticalization, therapeutic exercise, gait restoration, locomotor retraining and secondary complication prevention.

Restorative gait robotics aim to improve the person’s own walking capacity. They are most relevant when residual voluntary control, sensory feedback and balance potential can be trained. This profile is more common in AIS C or AIS D injuries, especially when the lesion is incomplete and the patient can participate actively in stepping. Assistive exoskeletons may also produce training effects, but their immediate goal can be upright mobility, loading, exercise, participation or psychological benefit. In motor-complete thoracic SCI, the expectation of independent community ambulation should be cautious because walking speed, energy expenditure and environmental demands remain major barriers. The same device can therefore be restorative for one individual and mainly assistive or health-promoting for another. Figure 2 presents this taxonomy.

This distinction has practical implications for trial interpretation. A study that enrolls people with motor-complete thoracic SCI and measures independent walking may appear disappointing even if the intervention improves upright tolerance, exercise exposure or satisfaction. Conversely, a study in incomplete SCI may miss meaningful recovery if it records only device-assisted steps and not unassisted walking, balance confidence or endurance. The clinical question should be framed before the device or robotic strategy is chosen. Is the robot being used as a therapeutic stimulus, a mobility aid, an exercise device, a standing platform or a bridge toward conventional gait training? Each answer implies different candidates, safety thresholds, outcome measures and expectations.

### 4.2. Wearable Exoskeletons

Wearable exoskeletons such as ReWalk, Ekso and Indego have expanded the possibilities of overground robotic walking after SCI. They typically provide powered hip and knee movement, require assistive devices such as crutches or a walker and depend on user training for balance, weight shifts and safety. ReWalk has been widely studied in thoracic motor-complete SCI and is often discussed as a personal mobility exoskeleton. Ekso systems have been used in rehabilitation settings across a broad range of neurological conditions and SCI profiles. Indego is modular and emphasizes portability; because of its lightweight modular design, it may also be considered a potentially practical assistive option in selected users. The Hybrid Assistive Limb, known as HAL, is important because it uses bioelectrical signals to support voluntary movement intention and has been studied in incomplete SCI, although regulatory availability differs across countries [47,48,49,50].

Candidate selection is central. Wearable exoskeleton training requires adequate upper limb strength, trunk control, bone health, joint range, body dimensions and cardiovascular tolerance. Contraindications may include unstable fractures, severe osteoporosis, uncontrolled spasticity, pressure injuries, severe contractures, orthostatic intolerance or inability to follow instructions. Even when safety criteria are met, real-world use can be limited by donning and doffing time, supervision needs, transport, stairs, uneven terrain, falls risk, device maintenance and cost. These issues explain why clinic-based training outcomes may not translate directly into community ambulation. Table 2 summarizes representative lower limb systems and their practical implications [51,52,53,54].

Training intensity also differs across wearable systems. Some programs, including those provided with Ekso-NR, begin with sit-to-stand practice, static balance and weight shifting before progressing to straight walking, turns and variable surfaces. Others remain confined to highly supervised indoor sessions. These differences matter because exoskeleton walking is a complex motor task that requires the user to coordinate upper limb support, trunk alignment, device timing and environmental attention. Fatigue can appear in the shoulders and wrists even when the legs are powered by the device. This means that a walking distance achieved in a laboratory should not be equated with practical community mobility unless speed, terrain, supervision and recovery time are also considered.

Long-term adherence is therefore a practical outcome, not a secondary detail. Home-based or personal exoskeleton use may extend training dose beyond the clinic, but it also transfers part of the safety burden to users, caregivers and community therapists. Device abandonment can occur because of discomfort, shoulder or wrist fatigue, slow walking speed, setup burden, limited environmental accessibility, insufficient technical support or reimbursement barriers. Future studies should report not only walking distance and session number, but also sustained use, reasons for discontinuation, caregiver time, adverse events and whether device-assisted gains transfer to meaningful home or community activities.

### 4.3. Locomotor Robotic Systems

Locomotor robotic systems such as Lokomat use treadmill-based robotic guidance with body-weight support to deliver repeated stepping cycles. The therapeutic rationale is based on task-specific locomotor input, symmetrical practice, adjustable guidance and reduced therapist burden. Body-weight support systems can also be used without a robotic orthosis, and end-effector devices can reproduce stepping trajectories through distal foot plates. These approaches are attractive in inpatient and early outpatient rehabilitation because they allow individuals with weakness to practice stepping safely before overground gait is feasible [55,56]. They also make it possible to standardize intensity, duration and kinematics, although excessive guidance may reduce active participation if not adjusted carefully.

The main limitation of treadmill-based RAGT is that the practiced task may differ from real-world walking. Community ambulation requires variable speed, turning, obstacle negotiation, attention, endurance, balance reactions and environmental decision-making. Robotic stepping can support foundational components, but it should be embedded in a broader gait rehabilitation program that includes overground practice when feasible. In incomplete SCI, the combination of robotic stepping, strength training, balance training and task-specific overground practice is often more clinically plausible than reliance on one modality. Device-derived parameters, including guidance force and active contribution, may help clinicians progress training, but these metrics require standardization before they can guide prescription across centers [57,58,59].

Therapist input remains essential even when a robotic system provides the mechanical stepping pattern. The therapist adjusts body-weight support, monitors skin and autonomic responses, encourages active effort, manages spasticity and decides when to reduce guidance. The patient must learn to tolerate upright posture, interpret feedback and transfer gains to less constrained tasks. For this reason, RAGT should be considered a component of a multimodal locomotor program rather than a replacement for skilled rehabilitation. Its strongest role may be to make high-dose stepping feasible while preserving therapist attention for decision-making, cueing and individualized progression.

### 4.4. End-Effector Devices

End-effector devices represent a distinct category within lower limb robotic systems because they guide gait through distal footplates or trajectory-controlled stepping platforms rather than by controlling the entire limb through a joint-aligned exoskeleton. This design can support repetitive, task-specific stepping, gait-phase practice, proprioceptive input, balance-related stepping and locomotor training while reducing some of the alignment constraints associated with full exoskeleton systems. Early clinical descriptions and systematic reviews contextualized end-effector systems within RAGT for SCI and related neurological populations [60,61].

In clinical practice, end-effector devices may be especially relevant for incomplete SCI when overground stepping is not yet safe, reproducible or sufficiently intensive. Their distal-control architecture may facilitate repeated stepping trajectories and adjustable assistance, but it provides less direct control of hip, knee and pelvic motion than exoskeleton-based systems. Therapist supervision therefore remains essential to monitor trunk or pelvic compensations, adapt body-weight support and integrate gains into overground walking. Feasibility and SCI-specific studies, including pilot work on neurophysiological outcomes, suggest potential benefits for gait ability, lower limb strength, balance, proprioception and functional ambulation, but available evidence remains limited by small samples and heterogeneous protocols [62,63,64,65]. End-effector robots should therefore be considered as components of a multimodal locomotor program rather than as stand-alone substitutes for individualized gait rehabilitation.

### 4.5. Current Evidence and Limitations

The evidence for lower limb robotics is broader than for upper limb robotics, but it remains heterogeneous. Meta-analyses and systematic reviews suggest that RAGT may improve selected walking and functional outcomes in SCI, especially in incomplete lesions, but effects vary by outcome measure, time since injury, intervention dose and study design [66,67,68]. Exoskeleton studies show feasibility, high user interest and improvements in training performance over time. Walking speed, however, often remains below thresholds required for efficient community ambulation. This limitation is not trivial. A device that allows a person to walk in the clinic may still be impractical for independent daily mobility if speed, terrain adaptability and setup demands are unfavorable [69,70,71].

Secondary health benefits may be clinically important even when independent ambulation is not achieved. Standing, stepping and upright exercise can influence cardiometabolic load, spasticity, bowel function, pain, body image and participation. Yet these effects are not uniform and should not be overgeneralized. The strongest current message is that lower limb robotic systems are feasible and potentially valuable when selected for the right patient and goal. The evidence does not support a universal prescription model. High cost, limited access, staff training needs and reimbursement barriers further restrict implementation. Future trials should separate restorative gait outcomes from assistive mobility, exercise and non-motor outcomes, because each requires different metrics and different expectations [72,73,74,75].

The most defensible interpretation is that lower limb robotics can expand the rehabilitation menu, but cannot remove the physiological and environmental barriers that determine walking in SCI. Complete injuries may gain standing, exercise, bowel-related routines, spasticity relief, mood benefits or participation experiences without achieving independent walking. Incomplete injuries may gain more direct locomotor recovery, especially when robotic training is combined with overground practice and progressive reduction in assistance. These different benefit profiles should be communicated clearly to patients before training starts.

## 5. Beyond Motor Recovery: Sensory and Non-Motor Effects of Robotics

Robotic rehabilitation in SCI should not be evaluated only through motor recovery. Many individuals live with sensory impairment and secondary health conditions that strongly influence quality of life, participation and healthcare use. Spasticity, neuropathic pain, cardiovascular deconditioning, orthostatic intolerance, bowel dysfunction, bone loss, pressure injury risk, fatigue and psychological distress can be as disabling as the motor deficit itself [76,77,78]. Robotic technologies may influence these domains through repeated movement, upright loading, rhythmic afferent input, exercise intensity, social participation and perceived agency. The evidence is uneven, so each outcome should be interpreted according to standardized levels of certainty rather than inferred from device exposure alone.

Sensory effects deserve explicit consideration. Proprioceptive input, load-related afference, cutaneous stimulation from device interfaces and visual feedback may support body-schema updating and sensorimotor recalibration during robot-assisted therapy. Sensory preservation may also moderate response because patients with residual proprioception or cutaneous feedback can often use error information more effectively during active practice. However, sensory outcomes are rarely primary endpoints in SCI robotics studies, and changes in sensibility, proprioception, pain interference and embodiment are often measured inconsistently. Future protocols should include formal sensory examination and patient-reported sensory or body-perception measures when sensory recovery or sensory compensation is part of the therapeutic rationale.

Spasticity is one of the most frequently discussed non-motor targets. Repetitive stepping, stretching, reciprocal movement and upright loading may transiently reduce muscle tone in selected individuals, possibly through modulation of spinal excitability and reciprocal sensorimotor activation [79,80]. The effect is not uniform, and fatigue, pain, inappropriate speed or poor alignment may worsen spasms in some patients. For this reason, spasticity should be monitored before and after robotic sessions using clinician-rated and patient-reported measures. Recent dose-oriented evidence also suggests that wearable powered overground exoskeleton training may reduce spasticity in a dose-dependent manner, although these findings require prospective confirmation and should not be overgeneralized [81].

Pain outcomes require even greater caution. Neuropathic pain after SCI is biologically heterogeneous and may be influenced by sensory deafferentation, central sensitization, sleep, mood, physical conditioning and functional participation. Robotic training may help some individuals by increasing movement, changing attention, improving self-efficacy or reducing musculoskeletal overload from prolonged sitting. It may also aggravate pain if harnesses, orthoses, straps or repetitive loading irritate joints and soft tissues [82,83]. Robotics should therefore not be framed as an analgesic intervention, but as a possible component of a broader pain management strategy when mechanical triggers are avoided.

Cardiovascular, metabolic, bowel, bladder, bone and pressure-related outcomes should be monitored directly rather than inferred from step counts or training exposure alone. Exoskeleton walking can increase heart rate and oxygen consumption compared with sitting, although intensity varies widely and may remain moderate [84,85,86]. Upright posture, trunk movement and scheduled activity may influence bowel routine efficiency, whereas bladder effects are less consistently supported and should be discussed cautiously [87,88]. Robotic standing and stepping provide mechanical loading, but whether the dose is sufficient to preserve or restore bone mineral density remains uncertain [89]. Quality of life, participation, psychological and embodiment effects may be highly meaningful because upright interaction, visible progress and technological agency can influence self-efficacy, motivation and perceived identity [90,91,92,93]. Table 3 therefore classifies the strength of evidence as moderate, limited, preliminary or mechanistically plausible, avoiding stronger claims than the available data support.

## 6. Robotics Combined with Advanced Neurotechnologies

One possible future direction for robotic rehabilitation in SCI is the integration of robotic systems with adjunctive neurotechnologies, although this approach remains at an early stage of clinical validation. Functional electrical stimulation (FES) can activate paralyzed muscles, provide afferent input and increase metabolic demand. When combined with robotics, FES may reduce passive movement, increase active muscle recruitment and create a closer coupling between intention, stimulation and movement [94,95]. Hybrid systems that combine exoskeletons with FES are conceptually attractive because they can distribute work between external actuators and biological muscle. However, current evidence is still mainly derived from small, heterogeneous or proof-of-concept studies, and superiority over well-dosed robotic or conventional rehabilitation alone has not yet been established. The practical challenge is synchronization. Stimulation timing, fatigue, spasticity, skin tolerance and individualized muscle responses must be managed carefully.

Virtual reality and gamification can increase engagement and provide meaningful feedback during repetitive training. This is important because robotic therapy can otherwise become monotonous. A virtual environment can convert reaching, grasping or stepping into goal-directed tasks, while performance scores can support motivation and progression. In SCI, virtual feedback may also contribute to embodiment by linking attempted movement to visible consequences. However, gamification should not replace clinical reasoning. A game that increases repetitions without targeting a relevant activity may improve engagement but not necessarily function. The best designs are those that connect therapeutic tasks with real-world goals, such as reaching for objects, navigating obstacles or controlling posture during dual-task walking [96,97].

Brain–computer interfaces (BCIs) and brain–machine interfaces (BMIs) provide a more direct link between neural intention and robotic action. Experimental systems have enabled exoskeleton or orthotic control from cortical signals, and some multimodal training protocols combine electroencephalography, virtual reality, tactile feedback and robotic gait assistance [98,99,100]. These approaches are mechanistically compelling, but their clinical role remains preliminary because most evidence comes from experimental or highly specialized settings. These approaches are attractive because they link neural intention with robotic action, but their implementation remains technically demanding because of signal quality, calibration time, cognitive load, reliability, cost and specialized infrastructure requirements. Current evidence supports feasibility and scientific promise more strongly than routine clinical implementation.

Non-invasive brain stimulation may modulate cortical excitability and thereby increase responsiveness to robotic training. Transcranial direct current stimulation has been paired with robot-assisted arm training in chronic incomplete cervical SCI, with proof-of-concept evidence suggesting possible benefit [101]. Repetitive transcranial magnetic stimulation and spinal stimulation approaches are also being explored as ways to alter excitability and promote training-induced plasticity [102,103]. Pilot work in spinal cord lesions further supports the feasibility of pairing robot-assisted rehabilitation with non-invasive neuromodulation, although evidence remains preliminary and protocols require validation [104]. The general principle is to pair neuromodulation with task-specific practice so that excitability changes occur in a behaviorally relevant context. The optimal stimulation site, timing, dose and responder profile remain uncertain. Accordingly, these combined protocols should currently be interpreted as hypothesis-generating strategies rather than established therapeutic standards.

Artificial intelligence (AI) and machine learning are beginning to influence robotic rehabilitation through adaptive control, prediction of responders and personalized progression. Algorithms can use kinematic, electromyographic, physiological and clinical data to estimate performance, fatigue or assistance needs. In theory, this could support the transition from fixed protocols toward more adaptive rehabilitation models, but this potential requires prospective validation before routine clinical use [105,106,107]. The same data streams could support digital rehabilitation records, home monitoring and benchmarking across centers. These possibilities require rigorous validation, transparency and governance. A black-box model that changes assistance without understandable clinical logic may create safety and ethical concerns.

Clinical deployment of these systems also raises regulatory and ethical questions. AI-supported or closed-loop robotics require transparent performance metrics, cybersecurity safeguards, privacy-preserving data governance and clinician oversight of any algorithm-driven dose progression. Explainability is particularly important when assistance levels, progression rules or risk alerts are generated from multidimensional sensor streams. Without prospective validation, audit trails and bias monitoring, adaptive systems may amplify inequities or create safety responsibilities that are difficult to assign. These requirements should be considered part of clinical readiness rather than optional technical refinements.

**Figure 3:**
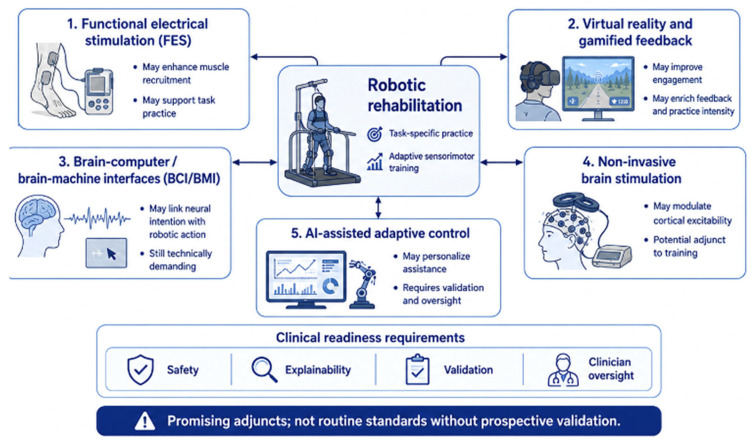
Adjunctive neurotechnologies that may be combined with robotic rehabilitation in spinal cord injury. These approaches include functional electrical stimulation, virtual reality, gamified feedback, brain–computer or brain–machine interfaces, non-invasive brain stimulation and artificial intelligence-assisted adaptive control. They should be interpreted as promising adjuncts rather than routine clinical standards.

Closed-loop rehabilitation should be interpreted broadly. It can mean a robot that adjusts assistance based on force output, a BCI that triggers movement based on motor intention, an FES system that responds to gait phase or an artificial intelligence platform that recommends dose progression. The common feature is feedback-driven personalization. Figure 3 illustrates how robotics can be combined with FES, virtual reality, BCI or BMI systems, non-invasive brain stimulation and AI-driven adaptation. The most clinically responsible position is cautious optimism. These combinations are promising and scientifically aligned with neuroplasticity, but many remain immature for routine prescription outside specialized centers [108,109,110,111,112,113].

Taken together, combined approaches integrating robotics with FES, virtual reality, BCI/BMI systems, non-invasive brain stimulation and AI-driven adaptation should be viewed as biologically plausible and potentially valuable adjuncts rather than validated routine standards. The available evidence remains largely preliminary, with frequent limitations related to small sample sizes, heterogeneous protocols, short follow-up, specialized settings and limited comparative data against well-dosed robotic-only or conventional rehabilitation programs. Their clinical use should therefore remain cautious, goal-directed and restricted to carefully selected patients, with predefined safety criteria, feasibility outcomes, regulatory oversight, explainable control logic and clinically meaningful endpoints rather than assumptions based on technological novelty alone.

## 7. A Clinical Framework for Personalized Robotic Rehabilitation in SCI

### 7.1. Stratification by SCI Severity

A severity-based framework for robotic rehabilitation should start with injury level and AIS grade because these variables shape both biological potential and clinical priorities. In the chronic stage, motor-complete or sensory-incomplete cervical SCI, usually AIS A or AIS B, often requires goals focused on assistance with activities of daily living, upper limb compensation, prevention of secondary complications, verticalization and quality of life. Robotic hand or arm devices may be used to increase practice opportunities, but expectations for major voluntary recovery should be cautious unless residual pathways are demonstrable. Wearable lower limb exoskeletons may be considered for standing and exercise if upper limb strength, trunk control and safety criteria are adequate, but caregiver and environmental demands must be explicit [114,115].

Complete thoracic SCI creates a different profile. Upper limb function is usually preserved, so lower limb exoskeletons may be feasible for upright exercise, standing practice, participation and secondary health benefits. The goal is often not independent community walking, although selected individuals may achieve supervised or limited personal use. The prescription should therefore define whether the primary target is exercise, bowel routine support, spasticity modulation, bone loading, psychological benefit or social participation. Ambulation metrics remain relevant, but they should not be the only markers of success. For many individuals with chronic complete thoracic SCI, the key question is whether the program provides benefits that justify time, cost and assistance needs [116,117]. For many individuals with motor-complete SCI, the most realistic exoskeleton-related outcomes may be supervised upright exercise, standing exposure, participation experience or secondary health benefits rather than independent community ambulation.

Incomplete cervical SCI, usually AIS C or AIS D, is one of the most important populations for upper limb restorative robotics. Residual descending motor control and sensory feedback can support task-oriented reach-to-grasp training, hand opening practice, robotic assistance and feedback-based motor learning. The goals often include dexterity, strength, coordination, self-care and independence in activities of daily living. The clinical prescription should identify whether the limiting factor is proximal weakness, distal hand control, sensory loss, spasticity, pain or endurance. This distinction determines whether a proximal arm robot, hand exoskeleton, electromyography-triggered device, soft glove or combined occupational task program is most appropriate [118,119].

Incomplete thoracic or lumbar SCI is the group in which restorative gait robotics may have the clearest rationale. If voluntary lower limb activation is present, robotic gait systems can provide high-dose stepping while preserving safety and repetition. Wearable exoskeletons may support overground task practice, confidence and balance progression. Treadmill-based systems may be valuable when overground walking is not yet safe. The expected targets include gait restoration, endurance, balance, neuroplasticity and community mobility when feasible. Time since injury matters. Early training may exploit spontaneous recovery and heightened plasticity, while chronic training may focus on motor relearning, conditioning and prevention of deconditioning [120,121].

Severity stratification should be understood as a starting point rather than a substitute for individualized assessment. Two people with the same AIS grade can differ in sensory preservation, autonomic tolerance, spasticity, hand dominance, cognition, pain and social support. These differences may completely change device suitability. The practical advantage of a severity-based framework is that it prevents clinicians from applying the same expectation to all patients. It encourages a distinction between restitution, substitution, conditioning and participation. It also makes it easier to explain why a person may receive a robot for exercise or upright experience rather than for walking recovery.

A further implication is that contraindications should be treated as modifiable or non-modifiable rather than as a simple exclusion list. Limited range of motion, deconditioning or orthostatic intolerance may improve with preparation. Severe osteoporosis, unstable skin or unsafe cognition may require alternative strategies. This distinction allows clinicians to create a pathway toward robotic use when appropriate while avoiding unsafe enthusiasm.

### 7.2. Stratification by Rehabilitation Goals

Goal-based stratification complements severity stratification. Hand dexterity should direct clinicians toward upper limb exoskeletons, end-effector systems, soft robotic gloves or electromyography-triggered hand assistance. Upright mobility should lead to wearable exoskeleton evaluation, provided that safety criteria are met. Recovery-oriented goals should prioritize assist-as-needed robotics, high-repetition task practice and active participation. Cardiovascular conditioning may justify overground exoskeletons, intensive RAGT or FES-cycling combinations. Spasticity management may justify repetitive gait robotics or upright stepping when clinically appropriate. Community ambulation requires the strictest scrutiny because it depends on speed, endurance, balance, device usability, environment and caregiver availability [122,123].

A personalized prescription should define the expected therapeutic target before the intervention begins. For example, a patient may train with an exoskeleton to increase walking independence, improve exercise tolerance, reduce sitting time or enhance participation. These are distinct targets and should be measured differently. Walking independence may require the 10-Meter Walk Test, 6-Minute Walk Test, Walking Index for Spinal Cord Injury, balance tests and real-world step counts. Exercise conditioning may require heart rate, perceived exertion and metabolic metrics. Participation may require goal attainment, community activity and satisfaction. Compared with existing locomotor practice guidance, the present framework adds a robotic prescription layer that links AIS grade, injury level, residual function, safety screening, device choice and outcome monitoring [124,125] (see Table 4).

Residual function is the operational bridge between severity and device selection. A person with trace hip activation but poor balance may need treadmill-based robotic stepping before overground exoskeleton training. A person with strong shoulder flexion but weak hand opening may need a distal device rather than proximal gravity support. A person with complete thoracic SCI and excellent upper limb strength may be a candidate for supervised exoskeleton walking, while a person with severe osteoporosis may not be safe despite strong motivation. The framework should therefore be applied as a clinical reasoning aid, not as a validated algorithm or checklist that automatically assigns devices [126].

Goal stratification should be revisited throughout rehabilitation. A person who initially uses a device for verticalization may later train for endurance, or a patient who begins with dexterity practice may shift toward compensatory assistance when recovery plateaus. This dynamic view is important because SCI rehabilitation is not a single episode. It is a long-term process in which goals change with medical stability, family roles, employment, equipment access and personal identity. Robotic prescription should therefore include review points, stopping rules and criteria for escalation or transition to another device [127].

Outcome domains must mirror the selected goal. A trial of hand robotics focused on feeding should not be judged only by grip strength. An exoskeleton program prescribed for cardiometabolic conditioning should record exercise intensity and tolerance rather than only walking independence. A program prescribed for participation should include goal attainment, satisfaction and real-world activity. This alignment is also important for reimbursement and health policy, because a device may appear ineffective if evaluated with endpoints that do not match the reason it was prescribed.

### 7.3. Proposed Prescription Matrix and Iterative Dose Adaptation

The proposed prescription matrix begins with injury level and AIS grade because these variables shape both biological potential and clinical priorities. Cervical injuries usually require explicit consideration of upper limb, hand function and activities of daily living, whereas thoracic and lumbar injuries more often shift the focus toward standing, gait, exercise and participation. AIS A/B profiles should be interpreted cautiously as motor-complete or sensory-incomplete categories rather than as a single complete-injury group, while AIS C/D profiles generally indicate incomplete injury with greater residual motor potential. Residual voluntary function should then be examined at the joint and task level, because this information is more clinically informative than global severity alone. Robotic therapy must match the movement that can be attempted, assisted, trained or substituted.

The clinician should then identify the dominant rehabilitation goal. A goal may be hand dexterity, ADL support, upright mobility, gait restoration, endurance, spasticity management, cardiometabolic conditioning, bowel routine support, participation or quality of life. Time since injury modifies expectations and dose. In early and subacute rehabilitation, robotics may be used to increase safe repetitions while recovery is evolving. In chronic SCI, robotics may still improve task performance, conditioning, participation or self-efficacy, but restorative claims should be individualized. Safety screening follows. Bone density, skin integrity, orthostatic tolerance, autonomic risk, spasticity, pain, range of motion, cognition, cardiopulmonary status and anthropometry must be assessed before exoskeleton use or intensive RAGT. A practical safety, contraindication and feasibility checklist for this screening step is provided in Supplementary Table S3. Environmental feasibility then determines whether the intervention is clinic-based, home-based, community-oriented or primarily experimental. The final step is outcome monitoring, which should be aligned with the selected goal [128]. Suggested goal-specific outcome domains, measurement tools, device-derived metrics and monitoring time-points are summarized in Supplementary Table S4.

**Figure 4:**
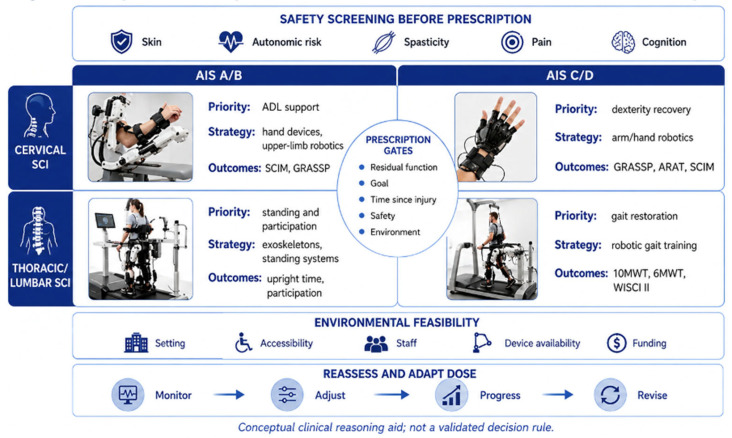
Simplified prescription matrix for personalized robotic rehabilitation in spinal cord injury. The matrix is intended as a conceptual clinical reasoning aid and not as a validated decision rule.

Figure 4 presents the proposed framework as a simplified 2 × 2 clinical reasoning matrix rather than as a rigid algorithm. The matrix organizes prescription logic according to injury level and AIS grade, and links each profile to dominant rehabilitation priorities, candidate robotic strategies and outcome domains. The central prescription gates emphasize residual voluntary function, primary rehabilitation goal, time since injury, safety screening and environmental feasibility. The lower reassessment loop indicates that robotic prescription should be iterative, response-guided and modifiable over time according to tolerance, progression, changing goals and clinically meaningful outcomes [129].

This framework has not yet undergone prospective validation. Its immediate role is to make prescription logic explicit and testable. Future validation should include Delphi consensus procedures, prospective cohort studies, multicenter pragmatic trials, implementation registries and responder-profiling studies. Such work should evaluate whether framework-guided prescription improves goal concordance, safety, adherence, outcome selection, cost-effectiveness and patient-reported relevance compared with device-availability-driven selection.

## 8. Challenges and Future Perspectives

The future of robotic rehabilitation in SCI depends on moving from access to intelligence. Many centers now have at least some robotic technology, but fewer have structured methods to identify responders, adjust dose or evaluate long-term value. Responder profiling will require integration of neurological classification, physiological measurements, imaging, device-derived metrics, autonomic status, patient goals and environmental context [130,131]. These measurements may include corticospinal excitability, electromyographic activation patterns, sensory preservation, gait variability, kinematic smoothness or measures of autonomic regulation. They should not be used to deny access prematurely, but they may help align expectations and personalize protocols.

AI may support personalization if it is used responsibly. Adaptive controllers can reduce assistance when the patient improves, detect fatigue and vary task difficulty. Predictive models may estimate which patients are likely to benefit from a given strategy. Digital platforms may support home robotics, telerehabilitation and remote monitoring. Yet these innovations raise ethical, regulatory and economic issues. Data privacy, cybersecurity, algorithmic bias, lack of explainability, device abandonment, unequal access and unclear reimbursement can undermine clinical benefit [132,133]. Home robotics is particularly attractive for long-term dose, but it requires safety, usability, technical support and caregiver integration. At present, AI-driven adaptive robotics should be considered a decision-support and dose-adjustment concept rather than an independently validated prescription tool.

Soft robotics may reduce barriers by creating lighter, more comfortable and more task-specific devices. Soft gloves, wearable actuators and textile-based systems could support daily hand assistance or high-repetition home practice without the bulk of rigid robots. For lower limbs, lighter exosuits and hybrid devices may improve portability and energy efficiency. The main challenge is to preserve enough mechanical support and reliability for people with severe weakness while maintaining comfort and affordability [134,135]. The distinction between assistive technology and therapeutic robotics may become less rigid as devices move into daily life.

Real-world implementation knowledge is equally important. Robotic programs require trained staff, maintenance, screening pathways, emergency procedures, outcome dashboards and realistic patient education. Cost-effectiveness studies are needed because the purchase price of a robot is only one part of implementation. Staff time, throughput, reimbursement, patient travel, caregiver involvement and long-term use determine value [136,137]. Pragmatic trials and multicenter registries could capture real-world outcomes better than small single-center studies. Equity should remain central. A robotic device or program that benefits only a small group with access to specialized centers may widen rehabilitation disparities [138,139].

From a real-world implementation perspective, the adoption of robotic rehabilitation in SCI also depends on costs, workforce training, organizational sustainability and territorial diffusion. Robotic programs require substantial upfront investment, dedicated spaces, maintenance contracts, device-specific staff training, safety procedures, patient throughput planning and reimbursement pathways. These requirements may limit diffusion outside highly specialized rehabilitation centers, particularly in rural, community-based or resource-limited services. Sustainable implementation should therefore be evaluated not only through technological performance or impairment-level outcomes, but also through pragmatic indicators such as the number of eligible patients, therapist time per session, donning and doffing time, equipment downtime, caregiver involvement, adverse-event preparedness, cost per clinically meaningful outcome and feasibility of transition to outpatient, home-based or community programs. Staff training should include neurological screening, fitting procedures, emergency release, fall prevention, skin protection, autonomic monitoring, fatigue and spasticity management, and criteria for progression or discontinuation. Without these organizational conditions, robotic technologies risk remaining high-visibility but low-reach interventions; with appropriate training, reimbursement models and stepped-care pathways, they may be integrated more realistically into specialized-center initiation, outpatient follow-up and selected supervised home or community use.

Future research should also improve the language used to report robotic interventions. Studies should describe not only device name and session duration, but assistance level, active contribution, progression rules, therapist input, adverse events, dropouts and home transfer. Standardized reporting would make trials more comparable and help clinicians reproduce protocols. Registries could complement randomized trials by capturing rare adverse events, durability, device abandonment and participation outcomes in real-world settings.

Economic evaluation will become unavoidable as robotic programs expand. A device that improves impairment but requires high staffing, expensive maintenance and limited throughput may be difficult to justify without clear patient selection. Conversely, a device with modest impairment effects may be valuable if it reduces caregiver burden, increases activity or prevents complications. Ethical implementation should consider not only cost, but also who gains access, who is excluded by anthropometric or cognitive criteria and whether rural or under-resourced patients can benefit from digital or home-based models.

Training and credentialing also need attention. Safe robotic practice requires knowledge of neurological examination, device mechanics, emergency release procedures, fall prevention, autonomic dysreflexia, skin protection and fatigue management. Programs should define who can screen, fit, supervise and progress each device. This is not administrative detail. It directly affects safety, reproducibility and patient trust. As devices become more portable, training standards will be needed for home caregivers and community therapists as well as specialized centers.

Long-term surveillance should also record why patients stop using devices, because abandonment can reflect poor fit, pain, fatigue, logistics, cost, stigma, limited support or a goal that changed after the initial prescription in everyday clinical reality over time.

This patient-first interpretation also makes outcome selection more coherent, because the same device can legitimately target impairment, activity, participation or prevention depending on the clinical profile.

A structured prescription conversation should therefore include expected benefits, realistic limitations, safety conditions, training burden and the measures that will define success for that individual.

Such transparency is especially important when high-cost technologies are introduced into rehabilitation pathways that already differ across regions, payers and levels of specialist expertise.

Personalization should also protect patients from therapeutic inflation, where the symbolic value of walking or advanced technology overshadows the practical value of independence, comfort and participation.

The most defensible robotic program is consequently one that can explain why a device was selected, why a given dose was chosen and why the outcome measures reflect the patient goal.

## 9. Limitations

This review has limitations. First, it is a narrative synthesis rather than a systematic review, and no formal risk-of-bias assessment was performed. Although the search strategy was structured and reported in the Supplementary Material, selection bias cannot be excluded. Second, the evidence base is heterogeneous across SCI level, AIS grade, time since injury, device class, intervention dose, control condition and outcome measure, which limits direct comparison across studies. Third, SCI-specific evidence remains limited for several technologies, and some mechanistic or implementation insights are extrapolated from stroke rehabilitation or broader neurorehabilitation cohorts. Fourth, sensory and non-motor outcomes are clinically important but often supported by preliminary, indirect or mechanistically plausible evidence rather than definitive trials. Finally, the proposed prescription framework has not yet been prospectively validated and should be interpreted as a structured clinical reasoning model that requires empirical testing.

## 10. Conclusions

Robotic rehabilitation has become an important component of contemporary SCI care, but its clinical value depends on appropriate prescription. Upper limb robots, lower limb exoskeletons, treadmill-based systems and hybrid neurotechnologies serve different purposes and should not be selected only by availability or novelty. Injury level, AIS grade, residual voluntary and sensory function, time since injury, safety and the person’s priorities must shape the therapeutic plan. The framework proposed in this review emphasizes severity-based clinical reasoning and recognizes that robotics may support neurorecovery in selected incomplete injuries while also providing exercise, verticalization, assistance, participation and selected non-motor benefits in other profiles. Current evidence supports feasibility and selected benefits, but remains heterogeneous and requires more SCI-specific trials. The next phase of the field should be defined by prospective framework validation, adaptive dosing, transparent outcome monitoring, explainable control systems and equitable implementation.

A mature robotic rehabilitation program should define success by whether the selected device advances a goal that is meaningful, safe, measurable and concordant with the patient’s neurological profile and lived priorities.

## Figures and Tables

**Table 1:** Representative upper limb robotic systems for spinal cord injury rehabilitation.

Robotic System/Citations	Device Type	Target Function	Most Relevant SCI Population	Training Principle	Clinical Strengths	Main Limitations
**Armeo Spring** [10,31,32,34]	Gravity-support upper limb exoskeleton	Shoulder, elbow, wrist and hand-oriented reaching tasks	Incomplete cervical SCI with residual proximal voluntary movement	Gravity-supported, high-repetition virtual task practice with graded assistance	Large three-dimensional workspace, task engagement and objective movement data	Requires some residual active movement; limited specificity for fine distal dexterity
**Armeo Power** [10]	Powered upper limb exoskeleton	Assisted shoulder, elbow and arm mobilization; active-assisted reaching	Severe cervical SCI with very limited active movement, including selected motor-complete or sensory-incomplete profiles when the target is assisted mobilization	Powered assistance, passive or active-assisted ROM training and task-oriented practice	Useful when gravity support alone is insufficient; may support ROM, pain control, spasticity management and early practice exposure	Risk of excessive passive participation; limited SCI-specific controlled evidence and need for careful parameter adjustment
**ALEX/ALEx-type upper limb exoskeleton** [10]	Robotic upper limb exoskeleton or bilateral arm-training system	Bilateral or unilateral arm training, reaching and coordinated upper limb movement	Complete or incomplete cervical SCI when bilateral practice, proximal control or assisted movement exposure is clinically relevant	Robotic assistance, symmetrical or asymmetrical bilateral training and task-oriented repetition	May support coordinated bilateral practice and structured high-dose training	Device-specific SCI evidence remains limited; requires careful positioning, ROM tolerance and goal selection
**Diego** [10]	Dynamic arm-weight support and robotic upper limb training system	Shoulder and elbow movement, reaching, workspace exploration and task simulation	Cervical SCI with partial proximal control or severe weakness requiring arm-weight support	Dynamic unloading, virtual feedback and high-repetition task-oriented reaching practice	Facilitates arm elevation and repeated goal-directed movements with adjustable support	Less specific for intrinsic hand function; clinical transfer depends on task integration
**Amadeo** [10]	Robotic hand and finger rehabilitation device	Finger flexion, extension, isolated finger movement and hand sensorimotor training	Cervical SCI with impaired finger movement, reduced hand opening or limited selective finger control	Passive, active-assisted or robot-assisted finger movement with sensor-based feedback	Highly specific for distal hand and finger training; useful when distal dexterity is the main target	Does not train proximal arm positioning; requires adequate hand fit, joint range and skin tolerance
**InMotion ARM, WRIST or HAND** [10,28,33]	End-effector robotic platform	Planar reaching, wrist control and selected hand movements	Incomplete cervical SCI requiring controlled repetitive practice	Robotic guidance, resistance and performance quantification	Precise kinematics and well-established motor-learning paradigm	Less ecological for complex ADL and bimanual tasks
**Hand of Hope** [10,28]	Electromyography-triggered hand exoskeleton	Hand opening, closing and grasp initiation	Cervical SCI with detectable voluntary forearm or hand activation	Intent-driven assistance coupled to EMG activity	Links motor intention to movement and may reinforce agency	Requires reliable EMG signals and appropriate hand range
**ReoGo** [10,28,34]	End-effector upper limb robot	Reaching trajectories and arm transport	Incomplete cervical SCI with proximal weakness or poor coordination	Guided reaching with adjustable assistance and visual feedback	Structured proximal arm practice and reproducible trajectories	Limited direct training of fine manipulation
**Gloreha and soft robotic glove systems** [10]	Wearable or semi-wearable hand robot/soft actuator glove	Finger flexion, extension and functional grasp-release assistance	Chronic cervical SCI with impaired grasp and preserved arm positioning capacity	Compliant assistance during object manipulation, grasp-release practice or home-oriented task training	Activity-oriented distal hand support, comfort and potential portability	Variable force output, fitting constraints and limited evidence for long-term neurorecovery

Abbreviations: ADL, activities of daily living; EMG, electromyography; ROM, range of motion; SCI, spinal cord injury.

**Table 2:** Representative lower limb robotic and exoskeleton systems used in spinal cord injury rehabilitation.

Robotic Approach/Citations	Examples	Training Environment	Primary Goal	Candidate SCI Profile	Potential Advantages	Practical Limitations
Wearable powered exoskeletons [45,46,47,48,51,53,54]	ReWalk, Ekso, Indego	Overground clinical or selected personal use	Upright mobility, exercise and gait practice	Thoracic SCI with adequate upper limb and trunk capacity, selected incomplete SCI	Standing, stepping, participation and cardiometabolic activation	Cost, supervision, speed, terrain limits and donning time
Bioelectrically assisted exoskeleton [47,48,49,50,53]	Hybrid Assistive Limb	Overground or treadmill-based programs in specialized centers	Voluntary intention-linked gait assistance	Incomplete SCI with detectable bioelectrical signals	May reinforce active participation and sensorimotor coupling	Availability, calibration and heterogeneous regulatory access
Treadmill robotic orthosis [17,18,49,51,52,53,54]	Lokomat	Treadmill with body-weight support	Repetitive locomotor input and gait restoration	Incomplete SCI or early gait training when overground practice is unsafe	High dose, symmetry and reduced therapist physical load	Transfer to community walking may be incomplete
Body-weight-support robotic systems [17,18,51,52,54]	Treadmill or overground body-weight-support systems, including Andago- or RYSEN-type systems	Clinical gait laboratory or therapy gym	Safe stepping, balance and progressive loading	Broad SCI profiles depending on assistance and therapist support	Flexible integration with manual cues and overground tasks	Labor intensive when not robotized
End-effector gait robots [48,53]	G-EO-type or footplate systems	Treadmill-like robotic stepping with distal control	Repetitive stepping trajectories and gait-phase practice	Incomplete SCI needing structured stepping without full exoskeleton alignment	Kinematic repetition and adjustable assistance	Less direct control of proximal joints
Hybrid robotics with FES [11,17,53]	Exoskeleton or gait robot plus FES	Specialized rehabilitation settings	Active muscle recruitment and robotic assistance	Incomplete or complete SCI with stimulable muscles and intact skin	Biological activation, afferent input and metabolic demand	Fatigue, timing complexity and stimulation tolerance

**Abbreviations:** FES, functional electrical stimulation; SCI, spinal cord injury.

**Table 3:** Sensory and non-motor domains potentially influenced by robotic rehabilitation in spinal cord injury.

Non-Motor Domain/Citations	Proposed Mechanism	Evidence Level	Most Relevant Robotic Approaches	Clinical Interpretation
Sensory effects [20,21,22,23,24,25,26,27,28]	Proprioceptive, load-related, cutaneous and visual feedback; body-schema updating	Mechanistically plausible; preliminary clinical evidence	RAGT, body-weight support, upper limb robotics, VR/BCI-linked systems	Measure sensory change directly; sensory preservation may moderate response
Spasticity [78,79,80,81]	Rhythmic afferent input, stretching, reciprocal stepping and active movement	Preliminary to moderate	Robotic gait training, exoskeleton walking, FES-hybrid stepping	May improve transiently in selected patients and should be monitored with tone and goal-based measures
Neuropathic pain [79,82,83,90,93]	Activity, mood, sleep, attention and possible modulation of sensory input	Limited	Exoskeleton training, upper limb robotics, multimodal programs	Potential indirect benefit, but pain may also worsen with pressure or fatigue
Cardiovascular and metabolic health [73,75,77,84,85,86,89]	Upright exercise, muscle pump activation and increased energy expenditure	Moderate for physiological load, limited for long-term outcomes	Overground exoskeletons, intensive RAGT, FES cycling plus robotics	Useful as part of structured exercise prescription rather than isolated exposure
Orthostatic tolerance [84,86,89]	Repeated verticalization and autonomic adaptation	Limited	Standing exoskeletons, body-weight support and gradual upright training	Monitor blood pressure, symptoms and injury level carefully
Bowel function [87,88]	Upright posture, trunk movement, stepping and increased activity	Preliminary	Exoskeleton walking and repetitive gait robotics	Promising domain, but protocols and measures need standardization
Bone health [75,89]	Mechanical loading and weight-bearing exposure	Indirect and limited	Standing frames, exoskeletons and robotic stepping	Dose may be insufficient for major bone effects in chronic severe osteoporosis
Pressure injury risk [78,91]	Reduced sitting time and pressure redistribution	Mechanistically plausible	Verticalization, exoskeleton standing and mobility programs	Requires skin checks because devices can create focal pressure
Quality of life and participation [56,59,78,92,93]	Agency, upright interaction, visible progress and social engagement	Preliminary to moderate	Wearable exoskeletons and goal-oriented upper limb robotics	Highly patient-specific and best captured by participation and satisfaction outcomes
Psychological and embodiment effects [26,57,92,93]	Body ownership, self-efficacy, motivation and perceived upright identity	Preliminary	BCI-linked systems, VR robotics and overground exoskeletons	Clinically meaningful but vulnerable to expectation bias

Abbreviations: BCI, brain–computer interface; FES, functional electrical stimulation; RAGT, robot-assisted gait training; VR, virtual reality.

**Table 4:** Proposed severity-based and goal-oriented clinical prescription framework for robotic rehabilitation in spinal cord injury.

SCI Profile/Citations	Dominant Clinical Priority	Residual Function Needed	Preferred Robotic Strategy	Expected Therapeutic Target	Main Caution	Suggested Outcome Domains
Cervical AIS A/B (motor-complete/sensory-incomplete) [3,4,8,29,30,31,32,35,36,37,38,39,40,41,42,43,44,114,115]	ADL assistance, upper limb support, verticalization and complication prevention	Task-specific residual activation if restorative training is intended	Assistive hand devices, upper limb robotics, selected exoskeleton standing	Assistance, practice dose, self-care support and upright exposure	Avoid overclaiming neurorecovery without residual pathways	SCIM, GRASSP, goal attainment, caregiver burden and QoL
Thoracic AIS A/B (motor-complete/sensory-incomplete) [3,4,45,46,47,51,55,56,57,58,59,66,67,68,69,70,71,72,78,84,85,86,87,88,116,117]	Standing, exercise, bowel and spasticity management, participation	Adequate upper limb strength, trunk control and safety profile	Wearable exoskeletons, body-weight support and exercise-oriented robotics	Verticalization, conditioning and participation	Community ambulation may remain limited by speed and assistance needs	6MWT, exertion, bowel routine, spasticity, participation and satisfaction
Cervical AIS C/D [3,4,29,30,31,32,33,34,35,36,37,38,39,40,41,42,43,44,118,119]	Dexterity recovery, reach-to-grasp control and ADL independence	Voluntary proximal or distal activation and trainable attention	Upper limb exoskeletons, end-effectors, EMG-triggered hand systems	Motor relearning, coordination and task-oriented hand use	Device must match proximal versus distal impairment	GRASSP, ARAT, SCIM, kinematics and patient-specific ADL goals
Thoracic or lumbar AIS C/D [3,4,51,52,55,66,67,68,69,70,71,72,116,117,118,119,120,121,122,123]	Gait restoration, endurance, balance and community mobility when feasible	Residual lower limb motor control and safe weight bearing	Assist-as-needed RAGT, treadmill robotics, overground exoskeleton progression	Walking capacity, balance, endurance and neuroplasticity	Robotic stepping should transfer to overground task practice	10MWT, 6MWT, WISCI II, balance, falls and participation
Chronic complete SCI with stable health [45,46,47,56,59,70,75,78,84,85,86,87,88,124,125]	Exercise, upright participation and secondary complication management	Safety for verticalization and ability to use assistive devices	Supervised exoskeleton walking or standing-oriented robotics	Conditioning, standing tolerance, mood and engagement	Cost and time burden may exceed functional gain	Exertion, QoL, bowel function, spasticity and satisfaction
Early incomplete SCI [51,52,55,72,116,117,118,119,120,121,122,124,125]	High-dose recovery-oriented practice and prevention of deconditioning	Medical stability and ability to contribute actively	Treadmill RAGT, body-weight support, progressive overground robotics	Stepping intensity, motor recovery and confidence	Avoid excessive guidance that reduces active effort	Motor score, WISCI II, 10MWT, 6MWT and device-derived active participation

Abbreviations: 6MWT, 6-Minute Walk Test; 10MWT, 10-Meter Walk Test; ADL, activities of daily living; ARAT, Action Research Arm Test; EMG, electromyography; GRASSP, Graded Redefined Assessment of Strength, Sensibility and Prehension; QoL, quality of life; RAGT, robot-assisted gait training; SCI, spinal cord injury; SCIM, Spinal Cord Independence Measure; WISCI II, Walking Index for Spinal Cord Injury II.

## Data Availability

No new data were created or analyzed in this study. Data sharing is not applicable to this article.

## Supplementary Materials

The following supporting information can be downloaded at: https://www.mdpi.com/article/10.3390/brainsci16070732/s1, Supplementary Table S1: PubMed/MEDLINE search strategy by conceptual block; Supplementary Table S2: Evidence map of robotic rehabilitation domains in spinal cord injury; Supplementary Table S3: Safety, contraindications, and feasibility checklist before robotic rehabilitation prescription in spinal cord injury; Supplementary Table S4: Suggested out-come monitoring matrix for goal-oriented robotic rehabilitation in spinal cord injury.

## Abbreviations

The following abbreviations are used in this manuscript: 6MWT, 6-Minute Walk Test; 10MWT, 10-Meter Walk Test; ADL, activities of daily living; AI, artificial intelligence; ASIA, American Spinal Injury Association; AIS, ASIA Impairment Scale; ARAT, Action Research Arm Test; BCI, brain–computer interface; BMI, brain–machine interface; CPG, central pattern generator; EMG, electromyography; FES, functional electrical stimulation; GRASSP, Graded Redefined Assessment of Strength, Sensibility and Prehension; HAL, Hybrid Assistive Limb; QoL, quality of life; RAGT, robot-assisted gait training; ROM, range of motion; rTMS, repetitive transcranial magnetic stimulation; SCI, spinal cord injury; SCIM, Spinal Cord Independence Measure; tDCS, transcranial direct current stimulation; VR, virtual reality; WISCI II, Walking Index for Spinal Cord Injury II.

## References

[1] Lu Y., Shang Z., Zhang W., Pang M., Hu X., Dai Y., Shen R., Wu Y., Liu C., Luo T. (2024). Global incidence and characteristics of spinal cord injury since 2000–2021: A systematic review and meta-analysis. BMC Med..

[2] Liu Y., Yang X., He Z., Li J., Li Y., Wu Y., Manyande A., Feng M., Xiang H. (2023). Spinal cord injury: Global burden from 1990 to 2019 and projections up to 2030 using Bayesian age-period-cohort analysis. Front. Neurol..

[3] Rupp R., Biering-Sørensen F., Burns S.P., Graves D.E., Guest J., Jones L., Read M.S., Rodriguez G.M., Schuld C., Tansey-Md K.E. (2021). International Standards for Neurological Classification of Spinal Cord Injury: Revised 2019. Top. Spinal Cord Inj. Rehabil..

[4] Snider B.A., Eren F., Reeves R.K., Rupp R., Kirshblum S.C. (2023). The International Standards for Neurological Classification of Spinal Cord Injury: Classification Accuracy and Challenges. Top. Spinal Cord Inj. Rehabil..

[5] Anderson K.D. (2004). Targeting recovery: Priorities of the spinal cord-injured population. J. Neurotrauma.

[6] Simpson L.A., Eng J.J., Hsieh J.T., Wolfe D.L. (2012). Spinal Cord Injury Rehabilitation Evidence Scire Research Team. The health and life priorities of individuals with spinal cord injury: A systematic review. J. Neurotrauma.

[7] Lo C., Tran Y., Anderson K., Craig A., Middleton J. (2016). Functional Priorities in Persons with Spinal Cord Injury: Using Discrete Choice Experiments to Determine Preferences. J. Neurotrauma.

[8] Snoek G.J., IJzerman M.J., Hermens H.J., Maxwell D., Biering-Sorensen F. (2004). Survey of the needs of patients with spinal cord injury: Impact and priority for improvement in hand function in tetraplegics. Spinal Cord.

[9] Mekki M., Delgado A.D., Fry A., Putrino D., Huang V. (2018). Robotic Rehabilitation and Spinal Cord Injury: A Narrative Review. Neurotherapeutics.

[10] Morone G., de Sire A., Martino Cinnera A., Paci M., Perrero L., Invernizzi M., Lippi L., Agostini M., Aprile I., Casanova E. (2021). Upper Limb Robotic Rehabilitation for Patients with Cervical Spinal Cord Injury: A Comprehensive Review. Brain Sci..

[11] Tamburella F., Lorusso M., Tramontano M., Fadlun S., Masciullo M., Scivoletto G. (2022). Overground robotic training effects on walking and secondary health conditions in individuals with spinal cord injury: Systematic review. J. Neuroeng. Rehabil..

[12] He Y., Xu Y., Hai M., Feng Y., Liu P., Chen Z., Duan W. (2024). Exoskeleton-Assisted Rehabilitation and Neuroplasticity in Spinal Cord Injury. World Neurosurg..

[13] Park J.M., Kim Y.W., Lee S.J., Shin J.C. (2024). Robot-Assisted Gait Training in Individuals with Spinal Cord Injury: A Systematic Review and Meta-Analysis of Randomized Controlled Trials. Ann. Rehabil. Med..

[14] Nepomuceno P., Souza W.H., Pakosh M., Musselman K.E., Craven B.C. (2024). Exoskeleton-based exercises for overground gait and balance rehabilitation in spinal cord injury: A systematic review of dose and dosage parameters. J. Neuroeng. Rehabil..

[15] Edgerton V.R., Roy R.R. (2009). Robotic training and spinal cord plasticity. Brain Res. Bull..

[16] Minassian K., Hofstoetter U.S., Dzeladini F., Guertin P.A., Ijspeert A. (2017). The Human Central Pattern Generator for Locomotion: Does It Exist and Contribute to Walking?. Neuroscientist.

[17] Hubli M., Dietz V. (2013). The physiological basis of neurorehabilitation--locomotor training after spinal cord injury. J. Neuroeng. Rehabil..

[18] Dobkin B.H., Duncan P.W. (2012). Should body weight-supported treadmill training and robotic-assistive steppers for locomotor training trot back to the starting gate?. Neurorehabil Neural Repair..

[19] Marchal-Crespo L., Reinkensmeyer D.J. (2009). Review of control strategies for robotic movement training after neurologic injury. J. Neuroeng. Rehabil..

[20] Rossignol S., Frigon A. (2011). Recovery of locomotion after spinal cord injury: Some facts and mechanisms. Annu. Rev. Neurosci..

[21] Dietz V. (2010). Behavior of spinal neurons deprived of supraspinal input. Nat. Rev. Neurol..

[22] Courtine G., Sofroniew M.V. (2019). Spinal cord repair: Advances in biology and technology. Nat. Med..

[23] Nardone R., Höller Y., Brigo F., Seidl M., Christova M., Bergmann J., Golaszewski S., Trinka E. (2013). Functional brain reorganization after spinal cord injury: Systematic review of animal and human studies. Brain Res..

[24] Freund P., Weiskopf N., Ward N.S., Hutton C., Gall A., Ciccarelli O., Craggs M., Friston K., Thompson A.J. (2011). Disability, atrophy and cortical reorganization following spinal cord injury. Brain.

[25] Calderone A., Cardile D., De Luca R., Quartarone A., Corallo F., Calabrò R.S. (2024). Brain Plasticity in Patients with Spinal Cord Injuries: A Systematic Review. Int. J. Mol. Sci..

[26] Braun N., Debener S., Spychala N., Bongartz E., Sörös P., Müller H.H.O., Philipsen A. (2018). The Senses of Agency and Ownership: A Review. Front. Psychol..

[27] Turner D.L., Ramos-Murguialday A., Birbaumer N., Hoffmann U., Luft A. (2013). Neurophysiology of robot-mediated training and therapy: A perspective for future use in clinical populations. Front. Neurol..

[28] Balasubramanian S., Colombo R., Sterpi I., Sanguineti V., Burdet E. (2012). Robotic assessment of upper limb motor function after stroke. Am. J. Phys. Med. Rehabil..

[29] Lu X., Battistuzzo C.R., Zoghi M., Galea M.P. (2015). Effects of training on upper limb function after cervical spinal cord injury: A systematic review. Clin. Rehabil..

[30] Kloosterman M.G., Snoek G.J., Jannink M.J. (2009). Systematic review of the effects of exercise therapy on the upper extremity of patients with spinal-cord injury. Spinal Cord.

[31] Zariffa J., Kapadia N., Kramer J.L., Taylor P., Alizadeh-Meghrazi M., Zivanovic V., Willms R., Townson A., Curt A., Popovic M.R. (2011). Effect of a robotic rehabilitation device on upper limb function in a sub-acute cervical spinal cord injury population. IEEE Int. Conf. Rehabil. Robot..

[32] Zariffa J., Kapadia N., Kramer J.L., Taylor P., Alizadeh-Meghrazi M., Zivanovic V., Willms R., Townson A., Curt A., Popovic M.R. (2012). Feasibility and efficacy of upper limb robotic rehabilitation in a subacute cervical spinal cord injury population. Spinal Cord.

[33] Cortes M., Elder J., Rykman A., Murray L., Avedissian M., Stampas A., Thickbroom G.W., Pascual-Leone A., Krebs H.I., Valls-Sole J. (2013). Improved motor performance in chronic spinal cord injury following upper-limb robotic training. NeuroRehabilitation.

[34] Vanmulken D.A., Spooren A.I., Bongers H.M., Seelen H.A. (2015). Robot-assisted task-oriented upper extremity skill training in cervical spinal cord injury: A feasibility study. Spinal Cord.

[35] Francisco G.E., Yozbatiran N., Berliner J., OʼMalley M.K., Pehlivan A.U., Kadivar Z., Fitle K., Boake C. (2017). Robot-Assisted Training of Arm and Hand Movement Shows Functional Improvements for Incomplete Cervical Spinal Cord Injury. Am. J. Phys. Med. Rehabil..

[36] Frullo J.M., Elinger J., Pehlivan A.U., Fitle K., Nedley K., Francisco G.E., Sergi F., O’Malley M.K. (2017). Effects of Assist-As-Needed Upper Extremity Robotic Therapy after Incomplete Spinal Cord Injury: A Parallel-Group Controlled Trial. Front. Neurorobot..

[37] Kim J., Lee B.S., Lee H.J., Kim H.R., Cho D.Y., Lim J.E., Kim J.J., Kim H.Y., Han Z.A. (2019). Clinical efficacy of upper limb robotic therapy in people with tetraplegia: A pilot randomized controlled trial. Spinal Cord.

[38] Jung J.H., Lee H.J., Cho D.Y., Lim J.E., Lee B.S., Kwon S.H., Kim H.Y., Lee S.J. (2019). Effects of Combined Upper Limb Robotic Therapy in Patients with Tetraplegic Spinal Cord Injury. Ann. Rehabil. Med..

[39] Osuagwu B.A.C., Timms S., Peachment R., Dowie S., Thrussell H., Cross S., Shirley R., Segura-Fragoso A., Taylor J. (2020). Home-based rehabilitation using a soft robotic hand glove device leads to improvement in hand function in people with chronic spinal cord injury:a pilot study. J. Neuroeng. Rehabil..

[40] Cappello L., Meyer J.T., Galloway K.C., Peisner J.D., Granberry R., Wagner D.A., Engelhardt S., Paganoni S., Walsh C.J. (2018). Assisting hand function after spinal cord injury with a fabric-based soft robotic glove. J. Neuroeng. Rehabil..

[41] Singh H., Unger J., Zariffa J., Pakosh M., Jaglal S., Craven B.C., Musselman K.E. (2018). Robot-assisted upper extremity rehabilitation for cervical spinal cord injuries: A systematic scoping review. Disabil. Rehabil. Assist. Technol..

[42] Yozbatiran N., Francisco G.E. (2019). Robot-assisted Therapy for the Upper Limb after Cervical Spinal Cord Injury. Phys. Med. Rehabil. Clin. N. Am..

[43] Lozano-Berrio V., Alcobendas-Maestro M., Polonio-López B., Gil-Agudo A., de la Peña-González A., de Los Reyes-Guzmán A. (2022). The Impact of Robotic Therapy on the Self-Perception of Upper Limb Function in Cervical Spinal Cord Injury: A Pilot Randomized Controlled Trial. Int. J. Environ. Res. Public Health.

[44] Ho J.S., Ko K.S., Law S.W., Man G.C. (2023). The effectiveness of robotic-assisted upper limb rehabilitation to improve upper limb function in patients with cervical spinal cord injuries: A systematic literature review. Front. Neurol..

[45] Miller L.E., Zimmermann A.K., Herbert W.G. (2016). Clinical effectiveness and safety of powered exoskeleton-assisted walking in patients with spinal cord injury: Systematic review with meta-analysis. Med. Devices.

[46] Louie D.R., Eng J.J., Lam T. (2015). Spinal Cord Injury Research Evidence (SCIRE) Research Team. Gait speed using powered robotic exoskeletons after spinal cord injury: A systematic review and correlational study. J. Neuroeng. Rehabil..

[47] Lajeunesse V., Vincent C., Routhier F., Careau E., Michaud F. (2016). Exoskeletons’ design and usefulness evidence according to a systematic review of lower limb exoskeletons used for functional mobility by people with spinal cord injury. Disabil. Rehabil. Assist. Technol..

[48] Cheung E.Y.Y., Ng T.K.W., Yu K.K.K., Kwan R.L.C., Cheing G.L.Y. (2017). Robot-Assisted Training for People with Spinal Cord Injury: A Meta-Analysis. Arch. Phys. Med. Rehabil..

[49] Alashram A.R., Annino G., Padua E. (2021). Robot-assisted gait training in individuals with spinal cord injury: A systematic review for the clinical effectiveness of Lokomat. J. Clin. Neurosci..

[50] Fang C.Y., Tsai J.L., Li G.S., Lien A.S., Chang Y.J. (2020). Effects of Robot-Assisted Gait Training in Individuals with Spinal Cord Injury: A Meta-analysis. BioMed Res. Int..

[51] Hornby T.G., Reisman D.S., Ward I.G., Scheets P.L., Miller A., Haddad D., Fox E.J., Fritz N.E., Hawkins K., Henderson C.E. (2020). And the Locomotor CPG Appraisal Team. Clinical Practice Guideline to Improve Locomotor Function Following Chronic Stroke, Incomplete Spinal Cord Injury, and Brain Injury. J. Neurol. Phys. Ther..

[52] Labruyère R., van Hedel H.J. (2014). Strength training versus robot-assisted gait training after incomplete spinal cord injury: A randomized pilot study in patients depending on walking assistance. J. Neuroeng. Rehabil..

[53] Molteni F., Gasperini G., Cannaviello G., Guanziroli E. (2018). Exoskeleton and End-Effector Robots for Upper and Lower Limbs Rehabilitation: Narrative Review. PM R.

[54] Hayes S.C., James Wilcox C.R., Forbes White H.S., Vanicek N. (2018). The effects of robot assisted gait training on temporal-spatial characteristics of people with spinal cord injuries: A systematic review. J. Spinal Cord Med..

[55] Chang S.H., Afzal T., Berliner J., Francisco G.E., TIRR SCI Clinical Exoskeleton Group (2018). Exoskeleton-assisted gait training to improve gait in individuals with spinal cord injury: A pilot randomized study. Pilot Feasibility Stud..

[56] Gagnon D.H., Vermette M., Duclos C., Aubertin-Leheudre M., Ahmed S., Kairy D. (2019). Satisfaction and perceptions of long-term manual wheelchair users with a spinal cord injury upon completion of a locomotor training program with an overground robotic exoskeleton. Disabil. Rehabil. Assist. Technol..

[57] Manns P.J., Hurd C., Yang J.F. (2019). Perspectives of people with spinal cord injury learning to walk using a powered exoskeleton. J. Neuroeng. Rehabil..

[58] Tefertiller C., Hays K., Jones J., Jayaraman A., Hartigan C., Bushnik T., Forrest G.F. (2018). Initial Outcomes from a Multicenter Study Utilizing the Indego Powered Exoskeleton in Spinal Cord Injury. Top. Spinal Cord Inj. Rehabil..

[59] Juszczak M., Gallo E., Bushnik T. (2018). Examining the Effects of a Powered Exoskeleton on Quality of Life and Secondary Impairments in People Living with Spinal Cord Injury. Top. Spinal Cord Inj. Rehabil..

[60] Sale P., Franceschini M., Waldner A., Hesse S. (2012). Use of the robot assisted gait therapy in rehabilitation of patients with stroke and spinal cord injury. Eur. J. Phys. Rehabil. Med..

[61] Swinnen E., Duerinck S., Baeyens J.P., Meeusen R., Kerckhofs E. (2010). Effectiveness of robot-assisted gait training in persons with spinal cord injury: A systematic review. J. Rehabil. Med..

[62] Choi S., Kim S.W., Jeon H.R., Lee J.S., Kim D.Y., Lee J.W. (2020). Feasibility of Robot-Assisted Gait Training with an End-Effector Type Device for Various Neurologic Disorders. Brain Neurorehabil.

[63] Shin J.C., Jeon H.R., Kim D., Cho S.I., Min W.K., Lee J.S., Oh D.S., Yoo J. (2021). Effects on the Motor Function, Proprioception, Balance, and Gait Ability of the End-Effector Robot-Assisted Gait Training for Spinal Cord Injury Patients. Brain Sci..

[64] Calabrò R.S., Filoni S., Billeri L., Balletta T., Cannavò A., Militi A., Milardi D., Pignolo L., Naro A. (2021). Robotic Rehabilitation in Spinal Cord Injury: A Pilot Study on End-Effectors and Neurophysiological Outcomes. Ann. BioMed Eng..

[65] Shin J.C., Jeon H.R., Kim D., Min W.K., Lee J.S., Cho S.I., Oh D.S., Yoo J. (2023). Effects of end-effector robot-assisted gait training on gait ability, muscle strength, and balance in patients with spinal cord injury. NeuroRehabilitation.

[66] Hartigan C., Kandilakis C., Dalley S., Clausen M., Wilson E., Morrison S., Etheridge S., Farris R. (2015). Mobility Outcomes Following Five Training Sessions with a Powered Exoskeleton. Top. Spinal Cord Inj. Rehabil..

[67] Sale P., Russo E.F., Scarton A., Calabrò R.S., Masiero S., Filoni S. (2018). Training for mobility with exoskeleton robot in spinal cord injury patients: A pilot study. Eur. J. Phys. Rehabil. Med..

[68] McIntosh K., Charbonneau R., Bensaada Y., Bhatiya U., Ho C. (2020). The Safety and Feasibility of Exoskeletal-Assisted Walking in Acute Rehabilitation After Spinal Cord Injury. Arch. Phys. Med. Rehabil..

[69] Sale P., Russo E.F., Russo M., Masiero S., Piccione F., Calabrò R.S., Filoni S. (2016). Effects on mobility training and de-adaptations in subjects with Spinal Cord Injury due to a Wearable Robot: A preliminary report. BMC Neurol..

[70] Kressler J., Thomas C.K., Field-Fote E.C., Sanchez J., Widerström-Noga E., Cilien D.C., Gant K., Ginnety K., Gonzalez H., Martinez A. (2014). Understanding therapeutic benefits of overground bionic ambulation: Exploratory case series in persons with chronic, complete spinal cord injury. Arch. Phys. Med. Rehabil..

[71] Bach Baunsgaard C., Vig Nissen U., Katrin Brust A., Frotzler A., Ribeill C., Kalke Y.B., León N., Gómez B., Samuelsson K., Antepohl W. (2018). Gait training after spinal cord injury: Safety, feasibility and gait function following 8 weeks of training with the exoskeletons from Ekso Bionics. Spinal Cord.

[72] Tsai C.Y., Delgado A.D., Weinrauch W.J., Manente N., Levy I., Escalon M.X., Bryce T.N., Spungen A.M. (2020). Exoskeletal-Assisted Walking During Acute Inpatient Rehabilitation Leads to Motor and Functional Improvement in Persons with Spinal Cord Injury: A Pilot Study. Arch. Phys. Med. Rehabil..

[73] Escalona M.J., Brosseau R., Vermette M., Comtois A.S., Duclos C., Aubertin-Leheudre M., Gagnon D.H. (2018). Cardiorespiratory demand and rate of perceived exertion during overground walking with a robotic exoskeleton in long-term manual wheelchair users with chronic spinal cord injury: A cross-sectional study. Ann. Phys. Rehabil. Med..

[74] Alamro R.A., Chisholm A.E., Williams A.M.M., Carpenter M.G., Lam T. (2018). Overground walking with a robotic exoskeleton elicits trunk muscle activity in people with high-thoracic motor-complete spinal cord injury. J. Neuroeng. Rehabil..

[75] Karelis A.D., Carvalho L.P., Castillo M.J., Gagnon D.H., Aubertin-Leheudre M. (2017). Effect on body composition and bone mineral density of walking with a robotic exoskeleton in adults with chronic spinal cord injury. J. Rehabil. Med..

[76] Ramanujam A., Cirnigliaro C.M., Garbarini E., Asselin P., Pilkar R., Forrest G.F. (2018). Neuromechanical adaptations during a robotic powered exoskeleton assisted walking session. J. Spinal Cord Med..

[77] Kressler J., Domingo A. (2019). Cardiometabolic Challenges Provided by Variable Assisted Exoskeletal Versus Overground Walking in Chronic Motor-incomplete Paraplegia: A Case Series. J. Neurol. Phys. Ther..

[78] Baunsgaard C.B., Nissen U.V., Brust A.K., Frotzler A., Ribeill C., Kalke Y.B., León N., Gómez B., Samuelsson K., Antepohl W. (2018). Exoskeleton gait training after spinal cord injury: An exploratory study on secondary health conditions. J. Rehabil. Med..

[79] Stampacchia G., Rustici A., Bigazzi S., Gerini A., Tombini T., Mazzoleni S. (2016). Walking with a powered robotic exoskeleton: Subjective experience, spasticity and pain in spinal cord injured persons. NeuroRehabilitation.

[80] Yang A., Asselin P., Knezevic S., Kornfeld S., Spungen A.M. (2015). Assessment of In-Hospital Walking Velocity and Level of Assistance in a Powered Exoskeleton in Persons with Spinal Cord Injury. Top. Spinal Cord Inj. Rehabil..

[81] Pournajaf S., Morone G., Felzani G., Pellicciari L., Cocco E.S., Manzia C.M., Simoncini L., Calabrò R.S., Franceschini M. (2026). Wearable powered overground exoskeleton reduces dose-dependently the spasticity in individuals with spinal cord injury. Spinal Cord.

[82] Benson I., Hart K., Tussler D., van Middendorp J.J. (2016). Lower-limb exoskeletons for individuals with chronic spinal cord injury: Findings from a feasibility study. Clin. Rehabil..

[83] Guanziroli E., Cazzaniga M., Colombo L., Basilico S., Legnani G., Molteni F. (2019). Assistive powered exoskeleton for complete spinal cord injury: Correlations between walking ability and exoskeleton control. Eur. J. Phys. Rehabil. Med..

[84] Esquenazi A., Talaty M., Packel A., Saulino M. (2012). The ReWalk powered exoskeleton to restore ambulatory function to individuals with thoracic-level motor-complete spinal cord injury. Am. J. Phys. Med. Rehabil..

[85] Lonini L., Shawen N., Scanlan K., Rymer W.Z., Kording K.P., Jayaraman A. (2016). Accelerometry-enabled measurement of walking performance with a robotic exoskeleton: A pilot study. J. Neuroeng. Rehabil..

[86] Asselin P., Knezevic S., Kornfeld S., Cirnigliaro C., Agranova-Breyter I., Bauman W.A., Spungen A.M. (2015). Heart rate and oxygen demand of powered exoskeleton-assisted walking in persons with paraplegia. J. Rehabil. Res. Dev..

[87] Chun A., Asselin P.K., Knezevic S., Kornfeld S., Bauman W.A., Korsten M.A., Harel N.Y., Huang V., Spungen A.M. (2020). Changes in bowel function following exoskeletal-assisted walking in persons with spinal cord injury: An observational pilot study. Spinal Cord.

[88] Fineberg D.B., Asselin P., Harel N.Y., Agranova-Breyter I., Kornfeld S.D., Bauman W.A., Spungen A.M. (2013). Vertical ground reaction force-based analysis of powered exoskeleton-assisted walking in persons with motor-complete paraplegia. J. Spinal Cord Med..

[89] Evans N., Hartigan C., Kandilakis C., Pharo E., Clesson I. (2015). Acute Cardiorespiratory and Metabolic Responses During Exoskeleton-Assisted Walking Overground Among Persons with Chronic Spinal Cord Injury. Top. Spinal Cord Inj. Rehabil..

[90] Andresen S.R., Biering-Sørensen F., Hagen E.M., Nielsen J.F., Bach F.W., Finnerup N.B. (2016). Pain, spasticity and quality of life in individuals with traumatic spinal cord injury in Denmark. Spinal Cord.

[91] Jensen M.P., Molton I.R., Groah S.L., Campbell M.L., Charlifue S., Chiodo A., Forchheimer M., Krause J.S., Tate D. (2012). Secondary health conditions in individuals aging with SCI: Terminology, concepts and analytic approaches. Spinal Cord.

[92] Budd M.A., Gater J., Channell I. (2022). Psychosocial Consequences of Spinal Cord Injury: A Narrative Review. J. Pers. Med..

[93] Craig A., Tran Y., Middleton J. (2009). Psychological morbidity and spinal cord injury: A systematic review. Spinal Cord.

[94] Post M.W., van Leeuwen C.M. (2012). Psychosocial issues in spinal cord injury: A review. Spinal Cord.

[95] Halvorsen A., Pape K., Post M.W.M., Biering-Sørensen F., Mikalsen S., Hansen A.N., Steinsbekk A. (2021). Participation and quality of life in persons living with spinal cord injury in Norway. J. Rehabil. Med..

[96] Hu X., Feng J., Lu J., Pang R., Zhang A., Liu J., Gou X., Bai X., Wang J., Chang C. (2024). Effects of exoskeleton-assisted walking on bowel function in motor-complete spinal cord injury patients: Involvement of the brain-gut axis, a pilot study. Front. Neurosci..

[97] Nistor-Cseppento C.D., Gherle A., Negrut N., Bungau S.G., Sabau A.M., Radu A.F., Bungau A.F., Tit D.M., Uivaraseanu B., Ghitea T.C. (2022). The Outcomes of Robotic Rehabilitation Assisted Devices Following Spinal Cord Injury and the Prevention of Secondary Associated Complications. Medicina.

[98] van der Scheer J.W., Goosey-Tolfrey V.L., Valentino S.E., Davis G.M., Ho C.H. (2021). Functional electrical stimulation cycling exercise after spinal cord injury: A systematic review of health and fitness-related outcomes. J. Neuroeng. Rehabil..

[99] Alashram A.R., Annino G., Mercuri N.B. (2022). Changes in spasticity following functional electrical stimulation cycling in patients with spinal cord injury: A systematic review. J. Spinal Cord Med..

[100] Mazzoleni S., Battini E., Rustici A., Stampacchia G. (2017). An integrated gait rehabilitation training based on Functional Electrical Stimulation cycling and overground robotic exoskeleton in complete spinal cord injury patients: Preliminary results. IEEE Int. Conf. Rehabil. Robot..

[101] Prasad S., Aikat R., Labani S., Khanna N. (2018). Efficacy of Virtual Reality in Upper Limb Rehabilitation in Patients with Spinal Cord Injury: A Pilot Randomized Controlled Trial. Asian Spine J..

[102] Donati A.R., Shokur S., Morya E., Campos D.S., Moioli R.C., Gitti C.M., Augusto P.B., Tripodi S., Pires C.G., Pereira G.A. (2016). Long-Term Training with a Brain-Machine Interface-Based Gait Protocol Induces Partial Neurological Recovery in Paraplegic Patients. Sci. Rep..

[103] López-Larraz E., Trincado-Alonso F., Rajasekaran V., Pérez-Nombela S., Del-Ama A.J., Aranda J., Minguez J., Gil-Agudo A., Montesano L. (2016). Control of an Ambulatory Exoskeleton with a Brain-Machine Interface for Spinal Cord Injury Gait Rehabilitation. Front. Neurosci..

[104] Naro A., Billeri L., Balletta T., Lauria P., Onesta M.P., Calabrò R.S. (2022). Finding the Way to Improve Motor Recovery of Patients with Spinal Cord Lesions: A Case-Control Pilot Study on a Novel Neuromodulation Approach. Brain Sci..

[105] Benabid A.L., Costecalde T., Eliseyev A., Charvet G., Verney A., Karakas S., Foerster M., Lambert A., Morinière B., Abroug N. (2019). An exoskeleton controlled by an epidural wireless brain-machine interface in a tetraplegic patient: A proof-of-concept demonstration. Lancet Neurol..

[106] Colucci A., Vermehren M., Cavallo A., Angerhöfer C., Peekhaus N., Zollo L., Kim W.S., Paik N.J., Soekadar S.R. (2022). Brain-Computer Interface-Controlled Exoskeletons in Clinical Neurorehabilitation: Ready or Not?. Neurorehabil Neural Repair..

[107] Rupp R. (2014). Challenges in clinical applications of brain computer interfaces in individuals with spinal cord injury. Front. Neuroeng..

[108] Yozbatiran N., Keser Z., Davis M., Stampas A., O’Malley M.K., Cooper-Hay C., Frontera J., Fregni F., Francisco G.E. (2016). Transcranial direct current stimulation (tDCS) of the primary motor cortex and robot-assisted arm training in chronic incomplete cervical spinal cord injury: A proof of concept sham-randomized clinical study. NeuroRehabilitation.

[109] Picelli A., Chemello E., Castellazzi P., Filippetti M., Brugnera A., Gandolfi M., Waldner A., Saltuari L., Smania N. (2018). Combined effects of cerebellar transcranial direct current stimulation and transcutaneous spinal direct current stimulation on robot-assisted gait training in patients with chronic brain stroke: A pilot, single blind, randomized controlled trial. Restor. Neurol. Neurosci..

[110] Simonetti D., Zollo L., Milighetti S., Miccinilli S., Bravi M., Ranieri F., Magrone G., Guglielmelli E., Di Lazzaro V., Sterzi S. (2017). Literature Review on the Effects of tDCS Coupled with Robotic Therapy in Post Stroke Upper Limb Rehabilitation. Front. Hum. Neurosci..

[111] Young M.J., Lin D.J., Hochberg L.R. (2021). Brain-Computer Interfaces in Neurorecovery and Neurorehabilitation. Semin. Neurol..

[112] Wardhana D.P.W., Maliawan S., Mahadewa T.G.B., Rosyidi R.M., Wiranata S. (2023). The Impact of Machine Learning and Robot-Assisted Gait Training on Spinal Cord Injury: A Systematic Review and Meta-Analysis. J. Clin. Med..

[113] Calderone A., Latella D., Bonanno M., Quartarone A., Mojdehdehbaher S., Celesti A., Calabrò R.S. (2024). Towards Transforming Neurorehabilitation: The Impact of Artificial Intelligence on Diagnosis and Treatment of Neurological Disorders. Biomedicines.

[114] Kim K.H., Jeong J.H., Ko M.J., Lee S., Kwon W.K., Lee B.J. (2024). Using Artificial Intelligence in the Comprehensive Management of Spinal Cord Injury. Korean J. Neurotrauma.

[115] Abbas G.H., Speksnijder C., Ramnarain D., Parmar C., Parmar A., Ahmad S., Pouwels S. (2025). AI-Driven Rehabilitation Robotics: Advancements in and Impacts on Patient Recovery. Cureus.

[116] Gil-Agudo Á., Megía-García Á., Pons J.L., Sinovas-Alonso I., Comino-Suárez N., Lozano-Berrio V., Del-Ama A.J. (2023). Exoskeleton-based training improves walking independence in incomplete spinal cord injury patients: Results from a randomized controlled trial. J. Neuroeng. Rehabil..

[117] Edwards D.J., Forrest G., Cortes M., Weightman M.M., Sadowsky C., Chang S.H., Furman K., Bialek A., Prokup S., Carlow J. (2022). Walking improvement in chronic incomplete spinal cord injury with exoskeleton robotic training (WISE): A randomized controlled trial. Spinal Cord.

[118] Yıldırım M.A., Öneş K., Gökşenoğlu G. (2019). Early term effects of robotic assisted gait training on ambulation and functional capacity in patients with spinal cord injury. Turk. J. Med. Sci..

[119] Tarnacka B., Korczyński B., Frasuńska J. (2023). Impact of Robotic-Assisted Gait Training in Subacute Spinal Cord Injury Patients on Outcome Measure. Diagnostics.

[120] Liu W., Chen J. (2024). The efficacy of exoskeleton robotic training on ambulation recovery in patients with spinal cord injury: A meta-analysis. J. Spinal Cord Med..

[121] Moriarty B., Jacob T., Sadlowski M., Fowler M., Rowan C., Chavarria J., Avramis I., Rizkalla J. (2024). The use of exoskeleton robotic training on lower extremity function in spinal cord injuries: A systematic review. J. Orthop..

[122] Liu S., Chen F., Yin J., Wang G., Yang L. (2025). Comparative efficacy of robotic exoskeleton and conventional gait training in patients with spinal cord injury: A meta-analysis of randomized controlled trials. J. Neuroeng. Rehabil..

[123] Nadorf F., Wright M.A., López-Matas H., Porras E., Carnicero-Carmona A., Hensel C., Franz S., Weidner N., Vidal J., Opisso E. (2024). User-centered design of a personal-use exoskeleton: A clinical investigation on the feasibility and usability of the ABLE Exoskeleton device for individuals with spinal cord injury to perform skills for home and community environments. Front. Neurosci..

[124] Charette C., Déry J., Blanchette A.K., Faure C., Routhier F., Bouyer L.J., Lamontagne M.E. (2023). A systematic review of the determinants of implementation of a locomotor training program using a powered exoskeleton for individuals with a spinal cord injury. Clin. Rehabil..

[125] Onate D., Hogan C., Fitzgerald K., White K.T., Tansey K. (2024). Recommendations for clinical decision-making when offering exoskeletons for community use in individuals with spinal cord injury. Front. Rehabil. Sci..

[126] Pei Y., Tobita M., Dirlikov B., Arnold D., Tefertiller C., Gorgey A. (2025). Consumer views of functional electrical stimulation and robotic exoskeleton in SCI rehabilitation: A mini review. Artif. Organs.

[127] de Seta V., Romeni S. (2025). Multimodal closed-loop strategies for gait recovery after spinal cord injury and stroke via the integration of robotics and neuromodulation. Front. Neurosci..

[128] Algaba-Vidoy M., Pérez-Nombela S., Megía-García Á., Montero-Pardo C., Redondo-Galán C., de Los Reyes-Guzmán A., Serrano-Muñoz D., Gómez-Soriano J., Del-Ama A.J., Moreno J.C. (2025). Exploring the feasibility of combining transcutaneous electrical spinal cord stimulation and overground robotic exoskeleton for gait rehabilitation in patients with spinal cord injury. Front. Neurol..

[129] Hu X., Li N., Pang M., Bai S., Mo J., Yao S., Lu Y., Huang M., Di J., Kang Y. (2026). Brain-Computer Interface-Controlled Exoskeleton Training for Lower-Limb Rehabilitation in Spinal Cord Injury: A Pilot Randomized Clinical Trial. Ann. Neurol..

[130] Li S., Gao S., Hu Y., Xu J., Sheng W. (2025). Brain-Computer Interfaces in Spinal Cord Injury: A Promising Therapeutic Strategy. Eur. J. Neurosci..

[131] Nijhawan M., Kataria C. (2024). Effect of Transcranial Direct Current Stimulation on Lower Extremity Muscle Strength, Quality of Life, and Functional Recovery in Individuals with Incomplete Spinal Cord Injury: A Randomized Controlled Study. Cureus.

[132] Xiang X.N., Zong H.Y., Ou Y., Yu X., Cheng H., Du C.P., He H.C. (2021). Exoskeleton-assisted walking improves pulmonary function and walking parameters among individuals with spinal cord injury: A randomized controlled pilot study. J. Neuroeng. Rehabil..

[133] Xiang X.N., Ding M.F., Zong H.Y., Liu Y., Cheng H., He C.Q., He H.C. (2020). The safety and feasibility of a new rehabilitation robotic exoskeleton for assisting individuals with lower extremity motor complete lesions following spinal cord injury (SCI): An observational study. Spinal Cord.

[134] Grasmücke D., Zieriacks A., Jansen O., Fisahn C., Sczesny-Kaiser M., Wessling M., Meindl R.C., Schildhauer T.A., Aach M. (2017). Against the odds: What to expect in rehabilitation of chronic spinal cord injury with a neurologically controlled Hybrid Assistive Limb exoskeleton. A subgroup analysis of 55 patients according to age and lesion level. Neurosurg. Focus.

[135] Cruciger O., Schildhauer T.A., Meindl R.C., Tegenthoff M., Schwenkreis P., Citak M., Aach M. (2016). Impact of locomotion training with a neurologic controlled hybrid assistive limb (HAL) exoskeleton on neuropathic pain and health related quality of life (HRQoL) in chronic SCI: A case study. Disabil. Rehabil. Assist. Technol..

[136] Sczesny-Kaiser M., Höffken O., Aach M., Cruciger O., Grasmücke D., Meindl R., Schildhauer T.A., Schwenkreis P., Tegenthoff M. (2015). HAL^®^ exoskeleton training improves walking parameters and normalizes cortical excitability in primary somatosensory cortex in spinal cord injury patients. J. Neuroeng. Rehabil..

[137] Aach M., Cruciger O., Sczesny-Kaiser M., Höffken O., Meindl R., Tegenthoff M., Schwenkreis P., Sankai Y., Schildhauer T.A. (2014). Voluntary driven exoskeleton as a new tool for rehabilitation in chronic spinal cord injury: A pilot study. Spine J..

[138] Wirz M., Zemon D.H., Rupp R., Scheel A., Colombo G., Dietz V., Hornby T.G. (2005). Effectiveness of automated locomotor training in patients with chronic incomplete spinal cord injury: A multicenter trial. Arch. Phys. Med. Rehabil..

[139] Cano-de-la-Cuerda R., Blázquez-Fernández A., Marcos-Antón S., Sánchez-Herrera-Baeza P., Fernández-González P., Collado-Vázquez S., Jiménez-Antona C., Laguarta-Val S. (2024). Economic Cost of Rehabilitation with Robotic and Virtual Reality Systems in People with Neurological Disorders: A Systematic Review. J. Clin. Med..

