# Supplementary material for: Robotic Rehabilitation in Spinal Cord Injury: Neurophysiological Basis and Severity-Based Clinical Framework
Source: Brain Sci. 2026 Jul 11;16(7):732. doi: 10.3390/brainsci16070732 (PMC13406644; doi:10.3390/brainsci16070732)
Supplement: Supplementary file 1 [file brainsci-16-00732-s001.zip › brainsci-4397346-supplementary.pdf]

Supplementary Material S1-S4

These supplementary tables support a clinically oriented narrative review. They provide a structured PubMed/MEDLINE search strategy, a domain-level evidence map, a safety and feasibility checklist, and a goal-oriented outcome monitoring matrix for robotic rehabilitation across tetraplegia, paraplegia, complete and incomplete spinal cord injury profiles.

Supplementary Table S1. PubMed/MEDLINE search strategy by conceptual block. The final PubMed/MEDLINE search was conducted on 28 May 2026. Search strings were organized into conceptual blocks covering core spinal cord injury robotic rehabilitation, upper limb robotics, lower limb exoskeletons and robot-assisted gait training (RAGT) systems, sensory and non-motor outcomes, advanced neurotechnology combinations, artificial intelligence-driven adaptive and closed-loop robotics, and safety, feasibility, and implementation. The strategy intentionally covered tetraplegia, paraplegia, cervical SCI, thoracic/lumbar SCI and complete/incomplete profiles. It combined Medical Subject Headings and free-text terms to support a clinically oriented narrative synthesis rather than a systematic review; therefore, no formal risk-of-bias assessment or PRISMA flow diagram was performed.

Search block	PubMed/MEDLINE search string	Main concept captured	Use in manuscript
Core SCI robotic rehabilitation	("Spinal Cord Injuries"[MeSH Terms] OR "spinal cord injury" OR "spinal cord injuries" OR SCI OR tetraplegia OR paraplegia) AND ("Robotics"[MeSH Terms] OR robotics OR "robot-assisted rehabilitation" OR "robotic rehabilitation" OR exoskeleton OR exoskeletons OR "robot-assisted gait training" OR RAGT)	Overall evidence on robotic rehabilitation, exoskeleton-assisted training and robot-assisted gait training in SCI.	Background, rationale, device taxonomy and general evidence synthesis.
Upper limb robotics in cervical SCI	("Spinal Cord Injuries"[MeSH Terms] OR "spinal cord injury" OR "spinal cord injuries" OR tetraplegia) AND ("Upper Extremity"[MeSH Terms] OR "upper limb" OR "upper extremity" OR arm OR hand OR grasp OR dexterity) AND (robotics OR "robot-assisted therapy" OR exoskeleton OR exoskeletons OR "soft robotic glove" OR Armeo OR ReoGo OR InMotion OR "Hand of Hope" OR "electromyography-triggered")	Upper limb and hand robotic systems for tetraplegia and incomplete cervical SCI.	Section 3 and Table 1.
Lower limb exoskeletons and robotic gait systems	("Spinal Cord Injuries"[MeSH Terms] OR "spinal cord injury" OR "spinal cord injuries") AND ("Gait"[MeSH Terms] OR "Walking"[MeSH Terms] OR gait OR walking OR ambulation OR locomotor training) AND (exoskeleton OR exoskeletons OR "powered exoskeleton" OR ReWalk OR Ekso OR Indego OR "Hybrid Assistive Limb" OR HAL OR Lokomat OR "body-weight support" OR "body weight support" OR "end-effector" OR "robot-assisted gait training")	Wearable exoskeletons, treadmill robotic orthoses, body-weight-supported gait training and end-effector gait devices.	Section 4, Figure 2 and Table 2.
Sensory, non-motor outcomes and secondary health effects	("Spinal Cord Injuries"[MeSH Terms] OR "spinal cord injury" OR "spinal cord injuries") AND (robotics OR exoskeleton OR exoskeletons OR "robot-assisted rehabilitation" OR RAGT) AND (sensory OR proprioception OR proprioceptive OR afferent OR spasticity OR pain OR bowel OR cardiovascular OR cardiometabolic OR metabolic OR "quality of life" OR participation OR osteoporosis OR bone OR "pressure injury" OR orthostatic)	Potential sensory, non-motor and secondary health effects of robotic standing, walking, exercise and task-oriented training.	Section 5 and Table 3.
Advanced neurotechnology combinations	("Spinal Cord Injuries"[MeSH Terms] OR "spinal cord injury" OR "spinal cord injuries") AND (robotics OR exoskeleton OR exoskeletons OR "robotic rehabilitation") AND ("functional electrical stimulation" OR FES OR "virtual reality" OR gamification OR "brain-computer interface" OR BCI OR "brain-machine interface" OR BMI OR "transcranial direct current stimulation" OR tDCS OR "repetitive transcranial magnetic stimulation" OR rTMS OR neuromodulation)	Hybrid robotics combined with FES, VR, BCI/BMI and non-invasive brain stimulation.	Section 6 and Figure 3.
AI-driven adaptive and closed-loop robotics	("Spinal Cord Injuries"[MeSH Terms] OR "spinal cord injury" OR "spinal cord injuries" OR neurorehabilitation) AND ("Artificial Intelligence"[MeSH Terms] OR "artificial intelligence" OR AI OR "machine learning" OR "adaptive control" OR "closed-loop" OR "digital rehabilitation") AND (robotics OR "robotic rehabilitation" OR exoskeleton OR exoskeletons OR "rehabilitation robotics")	Adaptive control, machine learning, digital rehabilitation and data-driven personalization.	Sections 6 and 8.
Safety, feasibility and implementation	("Spinal Cord Injuries"[MeSH Terms] OR "spinal cord injury" OR "spinal cord injuries") AND (exoskeleton OR exoskeletons OR robotics OR "robot-assisted rehabilitation") AND (safety OR feasibility OR usability OR implementation OR cost OR costs OR accessibility OR "clinical decision" OR prescription OR contraindication OR contraindications)	Safety screening, feasibility, usability, implementation barriers, decision-making and cost/access considerations.	Sections 7 and 8, Figure 4 and Tables 4, S3 and S4.

Supplementary Table S2. Evidence map of robotic rehabilitation domains in spinal cord injury, including standardized interpretation of evidence strength where applicable.

Evidence domain	Main technology or approach	Candidate SCI population	Typical evidence type	Main outcome focus	Clinical interpretation	Key references
Upper limb robotics in cervical SCI	Arm exoskeletons, end-effector reaching robots, EMG-triggered hand devices and soft gloves.	Predominantly cervical SCI and tetraplegia, especially incomplete lesions with residual proximal or distal activation.	Pilot trials, feasibility studies, controlled studies and SCI-specific reviews.	Reach, grasp, hand opening, dexterity, ADL performance, kinematics and self-perceived upper limb function.	Evidence supports feasibility and selected functional signals, but larger SCI-specific trials with long-term ADL and participation outcomes are needed.	[28-43]
Wearable lower limb exoskeletons	ReWalk-, Ekso- and Indego-type powered exoskeletons and portable overground walking systems.	Thoracic motor-complete SCI with adequate upper limb capacity and selected incomplete SCI.	Systematic reviews, pilot trials, multicenter feasibility studies and observational cohorts.	Standing, overground walking, gait speed, walking distance, exercise exposure, satisfaction and secondary health effects.	Useful for upright mobility, exercise and participation in selected candidates, but community ambulation remains limited by speed, terrain, supervision and cost.	[44-47,54-64,107-116]
Treadmill and body-weight-supported robotic gait training	Lokomat-like robotic orthoses, body-weight support and treadmill-based stepping systems.	Incomplete SCI, early gait retraining when overground practice is unsafe and selected chronic cases.	Randomized trials, meta-analyses and clinical practice guidance.	Stepping dose, symmetry, gait restoration, walking capacity, endurance and therapist workload.	Most defensible as a component of multimodal gait rehabilitation rather than a stand-alone substitute for overground practice.	[48-53,59-61,107-113,129]
End-effector and footplate gait devices	G-EO-type or footplate-guided robotic gait systems and distal stepping platforms.	Incomplete SCI needing structured stepping without full exoskeleton alignment.	Narrative reviews, device-specific studies and gait-technology reviews.	Repetitive stepping trajectories, gait-phase practice, kinematic repetition and adjustable assistance.	May provide high-repetition locomotor practice, but proximal joint control and transfer to real-world gait should be assessed carefully.	[52,53]
Bioelectrically assisted exoskeleton training	Hybrid Assistive Limb and intention-linked robotic gait assistance.	Incomplete or chronic SCI with detectable bioelectrical signals and sufficient safety profile.	Pilot studies, observational studies and neurophysiological studies.	Voluntary intention-linked movement, gait parameters, cortical excitability, pain and quality of life.	Potentially attractive for active participation and sensorimotor coupling, but availability, calibration and regulatory access vary.	[125-128]
Hybrid robotics with FES	Exoskeleton or gait robot combined with FES or FES cycling.	Incomplete or complete SCI with stimulable muscles, intact skin and stimulation tolerance.	Systematic reviews, integrated gait rehabilitation studies and exploratory studies.	Muscle recruitment, afferent input, metabolic demand, spasticity and gait practice.	Biologically appealing because it couples robotic alignment with active muscle recruitment, but timing, fatigue and skin tolerance require careful management.	[90-92,118,119]
VR and gamified robotic therapy	Virtual tasks, immersive scenarios, augmented feedback and motivational scoring systems linked to robotic training.	SCI patients undergoing upper limb or gait rehabilitation when cognition, vision and tolerance allow feedback-rich training.	Pilot randomized trials and technology integration studies.	Engagement, motivation, task salience, visuomotor feedback and functional task practice.	Useful when virtual tasks reinforce clinically relevant movement quality rather than only entertainment or repetition.	[88,89,93]
BCI/BMI-controlled robotics	Electroencephalography-based BCI, implanted BMI, robotic exoskeleton control and neural-intention interfaces.	Selected patients with SCI in specialized research or highly specialized clinical environments.	Proof-of-concept demonstrations, experimental protocols and reviews.	Neural intention decoding, embodiment, closed-loop control, sensorimotor contingency and possible neurological recovery.	Promising but not routine. Calibration, reliability, cost, cognitive load and implementation complexity remain major barriers.	[94-98,102,120,121]
Non-invasive brain stimulation with robotics	tDCS, rTMS and related neuromodulation paired with robotic or task-specific training.	Incomplete cervical or lower limb SCI in proof-of-concept or adjunctive protocols.	Pilot studies and narrative or mechanistic reviews.	Cortical excitability, motor learning, arm or gait performance and responsiveness to practice.	Should be interpreted as investigational or selectively adjunctive until stimulation parameters and responder profiles are clearer.	[99-101,122]
AI, adaptive control and closed-loop personalization	Machine learning, adaptive assistance, sensor-based feedback, digital rehabilitation and closed-loop robotic control.	SCI patients whose therapy can be monitored through kinematic, EMG, physiological or clinical data streams.	Systematic reviews, conceptual reviews and emerging AI-focused studies.	Assistance adaptation, prediction of responders, fatigue monitoring, dose progression and individualized rehabilitation planning.	Potentially transformative, but requires interpretability, validation, governance and protection against algorithmic bias.	[103-106,118]
Sensory and non-motor outcomes	Robotic standing, exoskeleton walking, RAGT, hybrid FES-robotics, upper limb robotics, VR-linked robotics and BCI-linked systems.	Broad SCI profiles depending on safety and clinical goal.	Pilot studies, observational studies, systematic reviews and mechanistic synthesis.	Proprioceptive and cutaneous feedback, spasticity, pain, bowel function, cardiovascular load, bone health, quality of life and participation.	Clinically relevant but uneven evidence. Sensory and non-motor domains should be measured prospectively using standardized terminology and not inferred from device use alone.	[66-89,123,126]
Implementation, safety and decision-making	Clinical screening pathways, user-centered device selection, cost/access planning and team-based robotic prescription.	All SCI candidates considered for robotic rehabilitation.	Implementation reviews, user-centered design studies, clinical recommendations and economic/organizational perspectives.	Feasibility, safety, accessibility, adherence, staffing, reimbursement and real-world usability.	Prescription should be transparent, patient-tailored and aligned with feasible goals rather than driven by device availability alone.	[114-116,130]

Supplementary Table S3. Safety, contraindications, and feasibility checklist before robotic rehabilitation prescription in spinal cord injury.

Safety or feasibility domain	What to assess before prescription	Why it matters clinically	Most relevant robotic approaches	Suggested action	Supporting references
Neurological level and AIS grade	Confirm neurological level, AIS grade, motor/sensory preservation and lesion completeness using standard classification.	Defines biological potential, target priorities and whether goals are restorative, assistive, compensatory or health-promoting.	All upper limb, lower limb and hybrid robotic approaches.	Document level/AIS before device selection and link prescription to goal hierarchy.	[3,4,116]
Residual voluntary function	Assess joint-specific activation, EMG detectability, voluntary control consistency, fatigue and task-level contribution.	Assist-as-needed and intention-driven systems require active participation or detectable signals.	Upper limb robotics, EMG-triggered hand systems, HAL-type systems, assist-as-needed RAGT and BCI/BMI systems.	Match device assistance to residual control. Avoid fully passive use when the intended mechanism is motor learning.	[17-19,34-39,99,125-128]
Bone density and fracture risk	Review fracture history, chronicity, severe osteoporosis risk, weight-bearing tolerance and relevant imaging where indicated.	Exoskeleton walking, standing and robotic loading may create fracture risk in severe disuse osteoporosis.	Wearable exoskeletons, standing systems, robotic stepping and body-weight-supported gait training.	Screen high-risk patients and consider graded preparation or alternative non-loading approaches when risk is unacceptable.	[68,81,116]
Skin integrity and pressure tolerance	Inspect skin, scars, pressure injury risk areas, orthotic contact sites, sensation and tolerance to straps or harnesses.	Robotic interfaces can create focal pressure, friction or unnoticed tissue stress, especially with sensory loss.	Exoskeletons, upper limb orthoses, hand devices, harness-based gait systems and standing frames.	Perform pre/post-session skin checks and adjust interface fit, padding and session duration.	[69,74,83,89,116]
Orthostatic tolerance and autonomic risk	Assess blood pressure response, symptoms during verticalization, autonomic dysreflexia risk and cardiopulmonary status.	Cervical and high thoracic SCI can produce orthostatic intolerance or autonomic instability during upright training.	Exoskeleton standing/walking, body-weight support, standing frames and intensive gait robotics.	Use graded verticalization, monitor vital signs and stop training when autonomic warning signs appear.	[78,81,123,124]
Spasticity and spasms	Assess tone, clonus, trigger factors, spasm frequency, medication timing and whether movement reduces or aggravates tone.	Spasticity may improve transiently with rhythmic stepping, but may also worsen with pain, fatigue or poor alignment.	RAGT, exoskeleton walking, FES-hybrid stepping and body-weight-supported training.	Monitor tone and spasms before/after sessions and adapt speed, range, assistance and fatigue exposure.	[72,73,91]
Pain and musculoskeletal tolerance	Identify neuropathic pain, shoulder/wrist overload, joint pain, pressure pain and fatigue-related symptom worsening.	Robotic training may improve perceived function but can aggravate pain through straps, harnesses, crutches or repetitive loading.	Exoskeleton walking, upper limb robotics, hand orthoses and intensive gait systems.	Use pain monitoring, interface adjustment and progression rules. Avoid interpreting robotics as a primary analgesic intervention.	[72,74,75,82,126]
Range of motion and contractures	Assess hip, knee, ankle, shoulder, elbow, wrist and finger range required for device alignment and task practice.	Contractures can prevent safe fitting, alter kinematics and increase skin or joint stress.	All orthotic, exoskeleton and gait-training devices.	Treat modifiable restrictions before device use and avoid forced alignment when range is unsafe.	[50,116]
Upper limb and trunk capacity for exoskeleton use	Assess shoulder strength, hand grip, wrist tolerance, trunk control, transfers and ability to use crutches or walker when required.	Most wearable lower limb exoskeletons require upper limb support and safe weight shifting.	Wearable exoskeletons and overground robotic gait systems.	Require device-specific eligibility screening and document whether supervision or assistive devices are necessary.	[44-46,57,64,116]
Anthropometric compatibility and donning burden	Check height, weight, limb length, joint alignment, orthosis fit, donning/doffing time and caregiver needs.	Poor fit reduces safety and usability. Excessive donning burden can limit adherence or community translation.	Wearable exoskeletons, hand devices, soft gloves and orthotic robotic systems.	Perform trial fitting and document compatibility, time burden and support requirements before prescription.	[46,57,114-116]
Cognitive, communicative and behavioral feasibility	Assess understanding of instructions, attention, fear, motivation, fatigue, communication and ability to report discomfort.	Safe robotic training requires interaction with feedback, emergency instructions and symptom reporting.	All robotic approaches, particularly exoskeletons, BCI/BMI systems and home-based technologies.	Use supervised familiarization, simplified feedback and caregiver involvement when needed.	[56,94-98,116]
Environmental feasibility and access	Assess clinic/home space, transport, floor surfaces, caregiver support, staff training, maintenance, reimbursement and local expertise.	A technically suitable device may be impractical if the environment cannot support safe and sustained use.	Exoskeletons, home robotics, tele-rehabilitation platforms and long-term robotic programs.	Define whether use is inpatient, outpatient, home-based or community-oriented and include access barriers in shared decision-making.	[115,116,130]
FES-specific feasibility	Assess skin integrity, electrode tolerance, stimulable muscles, fatigue, spasticity response and stimulation timing.	FES adds biological activation but may be limited by fatigue, discomfort, skin issues and timing complexity.	Hybrid exoskeleton-FES systems, FES cycling plus robotics and FES-assisted gait training.	Start with individualized stimulation mapping and progress only if muscle response and skin tolerance are acceptable.	[90-92,118,119]
Data governance for adaptive or AI-driven systems	Assess data type, privacy, interpretability, clinician oversight, algorithm validation and device cybersecurity if applicable.	Closed-loop and AI-driven systems depend on data streams that may affect safety, bias and accountability.	AI-adaptive robotics, wearable sensors, home monitoring and digital rehabilitation platforms.	Use interpretable, supervised systems and avoid black-box progression without clinical review.	[103-106,118,130]

Supplementary Table S4. Suggested outcome monitoring matrix for severity-based and goal-oriented robotic rehabilitation in spinal cord injury.

Clinical goal	Candidate robotic strategy	Primary measurement target	Suggested clinical measures	Device-derived or physiological measures	Suggested timing	Supporting references
Hand dexterity and reach-to-grasp recovery	Upper limb exoskeletons, end-effector reaching robots, EMG-triggered hand assistance and soft robotic gloves.	Selective reach, grasp, release, manipulation and task-oriented hand use.	GRASSP, ARAT, SCIM self-care items, Goal Attainment Scaling and patient-specific ADL tasks.	Workspace, trajectory smoothness, repetitions, assistance level, force, EMG activation and movement time.	Baseline, mid-program, end of program and follow-up when feasible.	[28-43]
ADL support and caregiver burden	Assistive hand devices, soft gloves, upper limb robotics and selected compensatory robotic technologies.	Functional independence in feeding, grooming, object use, wheelchair-related tasks and caregiver assistance.	SCIM, goal attainment, caregiver time, task completion and satisfaction/usability scales.	Successful assisted tasks, setup time, active use time, assistance level and device abandonment.	Baseline, end of training, home-use review and follow-up.	[5-8,38-43]
Upright mobility and standing tolerance	Wearable exoskeletons, supported standing systems and body-weight-supported verticalization.	Standing tolerance, upright exposure, symptoms and feasibility of repeated use.	Standing duration, orthostatic symptoms, blood pressure, perceived exertion and adverse events.	Training minutes, sit-to-stand attempts, verticalization time, harness load and device assistance settings.	Every session for safety variables, with formal reassessment every 2-4 weeks.	[44-47,54-64,76-81]
Gait restoration and locomotor capacity	RAGT, body-weight-supported treadmill training, end-effector gait robots and progressive overground exoskeleton training.	Walking capacity, gait symmetry, stepping quality, balance and transfer to overground function.	10MWT, 6MWT, WISCI II, balance tests, falls record and functional ambulation measures.	Step count, guidance force, body-weight support percentage, active contribution, cadence and symmetry.	Baseline, every training block, discharge/end-point and follow-up.	[47-53,59-61,107-113,129]
Endurance and cardiometabolic conditioning	Overground exoskeleton walking, intensive RAGT, FES cycling plus robotics and hybrid gait programs.	Exercise tolerance, cardiovascular load, metabolic activation and fatigue resilience.	Heart rate, blood pressure, RPE, session duration, fatigue scales and exercise adherence.	Training volume, step count, estimated energy expenditure, assistance level and physiological sensor outputs.	Continuous or session-level monitoring, with formal reassessment every 4-6 weeks.	[66,78,81,90,123]
Spasticity management	Repetitive gait robotics, exoskeleton walking, FES-hybrid stepping and body-weight-supported rhythmic training.	Change in tone, spasm frequency and spasticity-related functional interference.	Modified Ashworth Scale, Penn Spasm Frequency Scale, patient-reported spasm burden and goal attainment.	Movement speed, range, repetitions, stimulation parameters and session-related tone changes.	Pre/post-session checks and periodic longitudinal review.	[72,73,91]
Pain monitoring and tolerability	Exoskeleton training, upper limb robotics, hand devices and multimodal robotic programs.	Neuropathic and musculoskeletal pain response, pressure discomfort and fatigue-related pain.	Numeric Rating Scale, pain interference measures, pain map, medication change and adverse-event log.	Strap pressure locations, training load, time in device, skin checks and fatigue measures.	Pre/post-session and follow-up if pain is a target outcome.	[72,74,75,82,126]
Bowel and secondary health routines	Exoskeleton walking, verticalization, repetitive gait robotics and activity-based programs.	Bowel routine efficiency, upright activity exposure and secondary complication monitoring.	Bowel diary, bowel routine time, constipation symptoms, SCIM bowel items and patient satisfaction.	Upright time, step dose, training frequency and activity exposure.	Weekly diary review and reassessment after 4-8 weeks.	[79,88,89]
Participation and quality of life	Wearable exoskeletons, goal-oriented upper limb robotics, home or community-oriented robotic programs and integrated multimodal care.	Participation, self-efficacy, satisfaction, identity, social role and quality of life.	Quality-of-life measures, participation scales, Goal Attainment Scaling, satisfaction/usability and community activity reports.	Real-world use time, training adherence, device use context and abandonment reasons.	Baseline, end of program and medium-term follow-up.	[55,56,58,84-87]
Neuroplasticity and responder tracking	Assist-as-needed robotics, BCI/BMI-linked devices, tDCS/rTMS paired with training, AI-adaptive robotics and sensorized platforms.	Neural excitability, voluntary activation, learning rate, sensorimotor coupling and response phenotype.	Motor scores, sensory examination, neurophysiological tests where available and clinical responder status.	EMG activation, kinematics, assistance reduction, error correction, sensor data and algorithm-derived progression markers.	Baseline and repeated blocks, ideally linked to predefined responder criteria.	[23-27,94-106,118-122]
Safety and adverse-event surveillance	All robotic strategies, especially exoskeletons, intensive gait systems, FES-hybrid training and home-based robotics.	Falls, skin injury, pain flare, autonomic symptoms, excessive fatigue, device malfunction and discontinuation.	Adverse-event log, skin inspection, blood pressure monitoring, falls record and therapy interruption reasons.	Emergency stops, device alarms, assistance settings, donning/doffing problems and sensor alerts.	Every session, with aggregate review at each program milestone.	[61,64,74,115,116,124,130]

Abbreviations: *6MWT, 6-Minute Walk Test; 10MWT, 10-Meter Walk Test; ADL, activities of daily living; AI, artificial intelligence; AIS, American Spinal Injury Association Impairment Scale; ARAT, Action Research Arm Test; BCI, brain-computer interface; BMI, brain-machine interface; BWS, body-weight support; EMG, electromyography; FES, functional electrical stimulation; GAS, Goal Attainment Scaling; GRASSP, Graded Redefined Assessment of Strength, Sensibility and Prehension; HAL, Hybrid Assistive Limb; HR, heart rate; MAS, Modified Ashworth Scale; NIBS, non-invasive brain stimulation; QoL, quality of life; RAGT, robot-assisted gait training; RPE, rating of perceived exertion; rTMS, repetitive transcranial magnetic stimulation; SCI, spinal cord injury; SCIM, Spinal Cord Independence Measure; tDCS, transcranial direct current stimulation; TMS, transcranial magnetic stimulation; VR, virtual reality; WISCI II, Walking Index for Spinal Cord Injury II.*
